# A haploinsufficiency restoration strategy corrects neurobehavioral deficits in *Nf1^+/–^* mice

**DOI:** 10.1172/JCI188932

**Published:** 2025-07-01

**Authors:** Su Jung Park, Jodi L. Lukkes, Ka-Kui Chan, Hayley P. Drozd, Callie B. Burgin, Shaomin Qian, Morgan McKenzie Sullivan, Cesar Gabriel Guevara, Nolen Cunningham, Stephanie Arenas, Makenna A. Collins, Jacob Zucker, JinHee Won, Abbi Smith, Li Jiang, Dana K. Mitchell, Steven D. Rhodes, Steven P. Angus, D. Wade Clapp

**Affiliations:** 1 Department of Pediatrics, Herman B Wells Center for Pediatric Research, Indiana University School of Medicine, Indianapolis, Indiana, USA.; 2 Research Institute for Korean Medicine, Pusan National University, Yangsan-si, South Korea.; 3 Department of Psychiatry,; 4 Medical Scientist Training Program,; 5 Department of Dermatology, and; 6 Department of Medical and Molecular Genetics, Indiana University School of Medicine, Indianapolis, Indiana, USA.; 7 Division of Hematology-Oncology, Department of Pediatrics, Indiana University School of Medicine, Riley Hospital for Children at Indiana University Health, Indianapolis, Indiana, USA.; 8 Indiana University Melvin and Bren Simon Comprehensive Cancer Center, Indianapolis, Indiana, USA.; 9 Department of Pharmacology and Toxicology, and; 10 Department of Biochemistry and Molecular Biology, Indiana University School of Medicine, Indianapolis, Indiana, USA.

**Keywords:** Genetics, Neuroscience, Genetic diseases, Neurodevelopment, Ubiquitin-proteosome system

## Abstract

Neurofibromatosis type 1 (NF1) is a genetic disorder caused by mutations of the *NF1* tumor suppressor gene resulting in the loss of function of neurofibromin, a GTPase-activating protein (GAP) for Ras. While the malignant manifestations of NF1 are associated with loss of heterozygosity of the residual WT allele, the nonmalignant neurodevelopmental sequelae, including autism spectrum disorder (ASD) and/or attention deficit hyperactivity disorder (ADHD) are prevalent morbidities that occur in the setting of neurofibromin haploinsufficiency. We reasoned that augmenting endogenous levels of WT neurofibromin could serve as a potential therapeutic strategy to correct the neurodevelopmental manifestations of NF1. Here, we used a combination of genetic screening and genetically engineered murine models to identify a role for the F-box protein FBXW11 as a regulator of neurofibromin degradation. Disruption of *Fbxw11*, through germline mutation or targeted genetic manipulation in the nucleus accumbens, increased neurofibromin levels, suppressed Ras-dependent ERK phosphorylation, and corrected social learning deficits and impulsive behaviors in male *Nf1^+/–^* mice. Our results demonstrate that preventing the degradation of neurofibromin is a feasible and effective approach to ameliorate the neurodevelopmental phenotypes in a haploinsufficient disease model.

## Abstract 2

The targeting of F-box protein FBXW11 to prevent neurofibromin degradation is an effective strategy to rescue haploinsufficient neurobehavioral phenotypes of *Nf1+/-* mice.

## Introduction

Neurofibromatosis type 1 (NF1) is an autosomal dominant genetic disorder that affects 1 out of 3,000 individuals worldwide and is characterized by a range of malignant and nonmalignant manifestations (1–4). *NF1* encodes neurofibromin, a GAP that negatively regulates the Ras signaling pathway. Loss of heterozygosity of the residual normal allele in the central or peripheral nervous system results in the development of slow-growing tumors that are a source of investigation in many laboratories. Less studied are therapies for the neurodevelopmental disorders, a source of morbidity in individuals with NF1. Fifty to seventy percent of children with NF1 will have attention deficit hyperactivity disorder (ADHD), resulting in substantial deficits related to school performance and social engagement (2, 4, 5). Similarly, there is a marked increase in social communication deficits, with up to 30% of patients with NF1 having symptoms compared with 1%–3% of the general population (6–8). Through negative regulation of the Ras pathway, neurofibromin modulates axon guidance, synaptic plasticity, neuronal differentiation, and glial function (9). Accordingly, disruption of these processes secondary to reduced neurofibromin protein expression likely underlies the diverse neurodevelopmental features observed in individuals with NF1 (1). We and others have shown that heterozygous loss of the murine homolog of *NF1* (*Nf1^+/–^*) renders mice haploinsufficient in a range of tissues (10). Importantly, prior work has demonstrated that this *Nf1^+/–^* murine model closely recapitulates the neurodevelopmental phenotypes exhibited by patients with NF1 (2, 6–8, 11–17) and can provide a platform for dissecting the cellular and biochemical underpinnings of these pathologic symptoms.

Pathogenic mutations in *NF1* can result in the accelerated degradation of neurofibromin secondary to dysregulated ubiquitination or posttranslational modifications (18). The mechanisms governing neurofibromin degradation by the ubiquitin proteasome pathway (UPP) remain incompletely understood, although specific factors have been previously identified (19–21). Cullin proteins, which act as scaffolds for multi-subunit E3 ubiquitin ligase complexes, have been shown to regulate the stability of neurofibromin (19), and prior work by Cichowski et al. demonstrated that neurofibromin is phosphorylated prior to its degradation (18, 22). A potential therapeutic strategy for the neurodevelopmental complications is to increase neurofibromin levels in affected tissues (23, 24). Thus, interfering with UPP-mediated degradation may restore the level of neurofibromin in the setting of haploinsufficiency. Given the evidence that neurofibromin levels are regulated by the UPP and phosphorylation status (18, 22), we reasoned that F-box proteins might act as substrate specificity factors for neurofibromin degradation by S-phase kinase–associated–Cullin 1 (SKP1-CUL1) F-box (SCF) ubiquitin ligase complexes (25, 26). Thus, we performed a genetic screen of F-box specificity factors, a family composed of nearly 70 members (27), and identify a role for the F-box protein FBXW11 in the regulation of neurofibromin degradation. FBXW11, a substrate-targeting subunit of the SCF E3 ligase complex, regulates the specificity and recruitment of phosphorylated substrates to the E3 ligase. Our findings demonstrate that disruption of *Fbxw11*, either through germline inactivation or targeted genetic manipulation in the nucleus accumbens, increases neurofibromin levels, reduces aberrant ERK phosphorylation, and corrects social learning deficits and impulsive behaviors in male *Nf1^+/–^* mice. Importantly, these results establish a potential paradigm for overcoming haploinsufficiency by blocking the UPP that could be explored in other diseases.

## Results

### FBXW11 mediates neurofibromin degradation.

**Figure 1:**
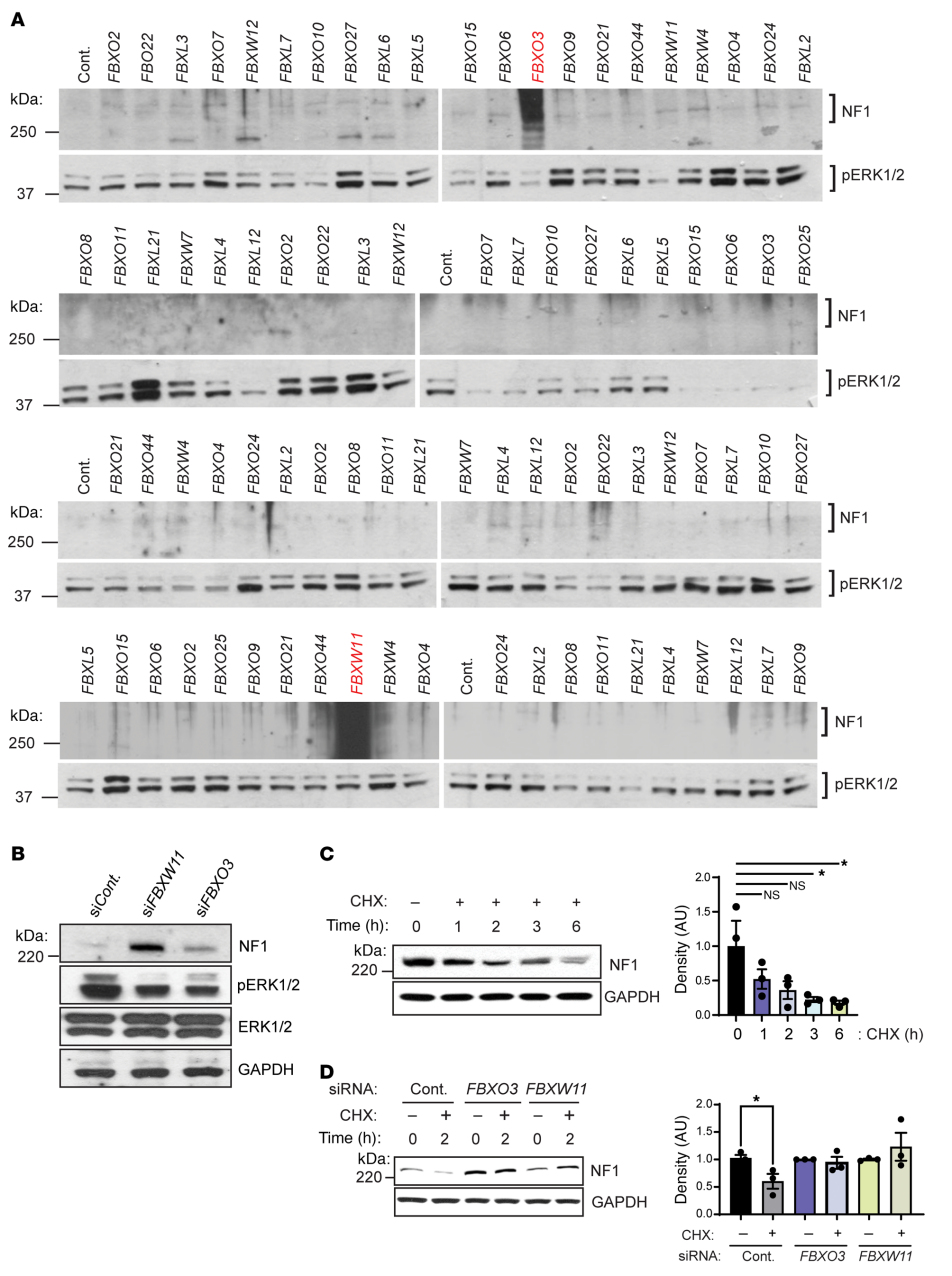
F-box proteins FBXW11 and FBXO3 are involved in the degradation of neurofibromin. (**A**) Control (Cont.) and F-box–specific siRNAs were transfected into human diploid fibroblasts. After 72 hours, lysates were prepared and used for immunoblotting to detect NF1 (top) and pERK1/2 (bottom doublet). Red font highlights increased neurofibromin levels in lysates prepared from wells containing siRNAs targeting *FBXW11* or *FBXO3*. An siRNA targeting *FBXW1A* (b*TrCP1*, *BTRC*) was not included. (**B**) HeLa cells were transfected with a control siRNA or with siRNAs targeting *FBXO3* or *FBXW11*. NF1, pERK1/2, and total ERK1/2 levels were analyzed 72 hours after transfection by immunoblotting. GAPDH was used as a loading control. (**C**) Left: CHX was added at a final concentration of 20 μg/mL to HeLa cells for the indicated durations prior to harvesting for immunoblotting to detect NF1. GAPDH was used as a loading control. Right: NF1 levels were quantified by densitometry relative to GAPDH. *P < 0.05, by 1-way ANOVA with Dunnett’s multiple-comparison test. Data indicate the mean ± SEM. (**D**) Left: HeLa cells were transfected with control siRNA or siRNA targeting *FBXO3* or *FBXW11*. Sixty-eight hours after transfection, cells were treated with CHX (20 μg/mL final concentration) for 2 hours prior to harvesting and immunoblotting to detect NF1. GAPDH was used as a loading control. Right: NF1 levels were quantified by densitometry relative to GAPDH. *P < 0.05 comparing siCont with or without CHX, by unpaired 2-tailed *t* test. Data indicate the mean ± SEM.

F-box proteins are substrate receptors that recruit phosphorylated substrates to the SCF ubiquitin-ligase complex (25, 26). Given the potential therapeutic relevance of identifying substrate receptors involved in neurofibromin degradation, we performed an unbiased F-box–wide RNAi library screen using human diploid fibroblasts. These cells were chosen for their diploid genotype, ease of transfection, and robust expression of neurofibromin. We found that transfection with siRNAs targeting *FBXW11/BTRC2* and *FBXO3* resulted in marked accumulation of neurofibromin in human fibroblasts (Figure 1A). While at least 2 siRNA sequences were utilized for each F-box protein screened, only 1 sequence targeting FBXO3 and FBXW11 resulted in a substantial increase of neurofibromin. Therefore, we validated these siRNA sequences from the library in a human mast cell line (LUVA), which is amenable to transfection and screening (Supplemental Figure 1; supplemental material available online with this article; https://doi.org/10.1172/JCI188932DS1). Mast cells represent a cell type for which we have established a role for neurofibromin haploinsufficiency in murine Nf1–associated tumorigenesis (28, 29). Using unique siRNA duplexes, FBXW11 and FBXO3 depletion by RNAi in HeLa cells also stabilized neurofibromin and suppressed constitutive phosphorylation of the Ras effectors ERK1 and ERK2 (Figure 1B).

To determine whether *FBXW11* or *FBXO3* knockdown could inhibit neurofibromin degradation, we next performed cycloheximide (CHX) chase experiments using HeLa cells (Figure 1, C and D). In control cells, neurofibromin levels were decreased 2 hours after treatment with CHX to block protein synthesis (Figure 1C). In contrast, neurofibromin levels were unchanged in HeLa cells transfected with *FBXW11* or *FBXO3* siRNA 2 hours following the CHX chase (Figure 1D). These observations provided further confirmation that depletion of FBXW11 or FBXO3 led to prolonged stability of neurofibromin protein.

### Overexpression of FBXW11 or FBXO3 reduces neurofibromin levels, whereas F-box-targeted drugs increase them.

**Figure 2:**
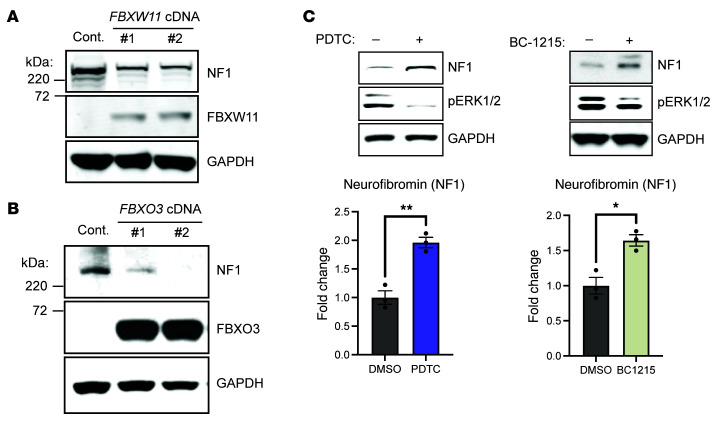
Neurofibromin levels affected by ectopic FBXW11 or FBXO3 expression or small-molecule F-box inhibitors. (**A** and **B**) HeLa cells transfected with an empty expression vector or with vectors containing either an FBXW11 cDNA or an FBXO3 cDNA were collected and lysed 48 hours later. NF1, FBXW11, and FBXO3 proteins were detected by immunoblotting, with GAPDH used as a loading control. (**C**) *Nf1^+/–^* MEFs were treated with the FBXW11 inhibitor PDTC (50 μM) or the FBXO inhibitor BC-1215 (20 μg/mL) for 6 hours prior to harvesting for immunoblotting. The lower panels represent densitometric analysis comparing NF1 levels with the GAPDH control. *P < 0.05 and **P < 0.01, by unpaired 2-tailed *t* test. Data indicate the mean ± SEM.

We next performed complementary experiments evaluating the effect of ectopic overexpression of either FBXW11 or FBXO3. Introduction of either F-box protein exogenously revealed a reduction in endogenous neurofibromin levels compared with controls (Figure 2, A and B). Consistent with these data, exposing haploinsufficient *Nf1^+/–^* murine embryonic fibroblasts (MEFs) to FBXW11 (pyrrolidine dithiocarbamate [PDTC]) (30–32) or FBXO3 (BC-1215) preclinical small-molecule inhibitors (33, 34) also increased neurofibromin protein levels and reduced ERK1/2 phosphorylation (Figure 2C). Taken together, these results suggest that FBXW11 and FBXO3 mediate neurofibromin degradation.

### FBXW11 preferentially interacts with neurofibromin isoform 1.

**Figure 3:**
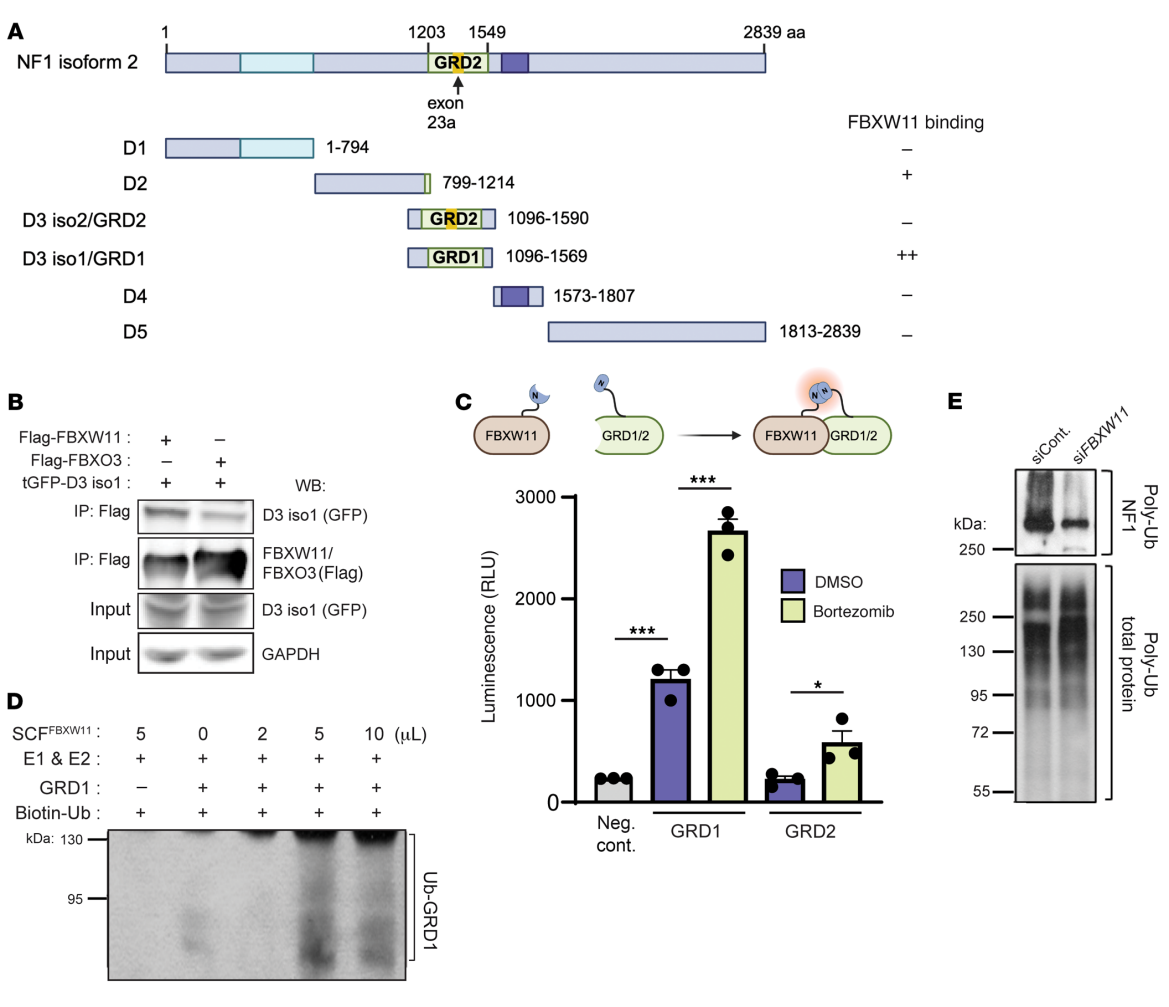
FBXW11 preferentially interacts with and polyubiquitinates the GRD of neurofibromin isoform 1. (**A**) Schematic map of the neurofibromin (NF1) interaction domains used to test interactions with FBXW11. Full-length NF1 was divided into the 6 indicated domains and subcloned into GFP-expressing plasmids. Flag-tagged FBXW11 and the various GFP-tagged NF1 subdomains were transfected into HEK293T cells. Six hours prior to harvesting, the cells were treated with 15 μM MG-132. Anti-Flag IP followed by immunoblotting was used to detect GFP-tagged NF1 co-IP with FBXW11. Relative binding activity as determined by immunoblotting is indicated. Image created in BioRender. Angus, S. (2025) https://BioRender.com/n28p603 (**B**) HEK293T cells were treated as in **A** to confirm the specific interaction of FBXW11 with the domain 3 (D3) fragment of the NF1 peptide, which encompasses the GRD of NF1 isoform 1 (GRD1), but not GRD2. (**C**) A NanoBiT complementation assay was performed after cotransfection of HEK293T cells with LgBiT-FBXW11 and SmBiT-GRD1 or GRD2 fusion proteins. Forty-five hours after transfection, the interaction was detected by luminescence. The negative control (neg. cont.) refers to luminescence values obtained from wells containing cells with LgbiT-FBXW11 and SmBiT-empty. Vehicle or the proteasome inhibitor bortezomib (1 mM) was included to enhance the interaction due to GRD1 accumulation. *P < 0.05 and ***P < 0.001, by unpaired 2-tailed *t* test. Data indicate the mean ± SEM. Image created in BioRender. Angus, S. (2025) https://BioRender.com/e61l197 (**D**) The SCF complex (SKP1, CUL1, RBX1, and Flag-FBXW11) was purified after cotransfection and subsequent Flag-IP (and Flag-peptide elution) from HEK293T cell lysates. Isolated GFP-GRD1 was incubated at 37°C for 60 minutes in the presence of E1 (100 nM), E2 (2 mM), Mg-ATP (5 mM), and ubiquitin (Ub) in the presence (+) or absence (–) of SCF^FBXW11^. Samples were then subjected to immunoblotting, and GRD1 ubiquitination was detected by GFP antibody and the smearing due to higher-molecular-weight species. (**E**) HEK293T cells were transfected with control or *FBXW11*-targeting siRNAs for 72 hours. A TUBEs assay was performed using agarose-TUBE2 to pull down total ubiquitinated protein. Immunoblotting was used to detect ubiquitin and NF1.

We next investigated biochemical interactions between different regions of neurofibromin with these F-box proteins by partitioning neurofibromin into 6 GFP-tagged peptides (domains D1, D2, D3 iso2, D3 iso1, D4, D5), which were expressed and observed at their predicted molecular weights by immunoblotting (Figure 3A and Supplemental Figure 2A). Alternative splicing of exon 23a within the catalytic GAP-related domain (GRD) of neurofibromin can produce 2 distinct isoforms: isoform 2 includes exon 23a in the GRD (D3 iso2/GRD2), whereas isoform 1 lacks exon 23a (D3 iso1/GRD1) (35, 36). Neurofibromin isoform 1, predominantly expressed in the adult brain (37), has 10-fold higher Ras-GAP activity than does isoform 2 (35, 38). Co-IP experiments to identify regions of neurofibromin that preferentially bound FBXW11 or FBXO3 revealed that FBXW11 interacted more strongly with D3 iso1/GRD1, whereas FBXO3 preferentially interacted with D3 iso2/GRD2 (Figure 3B and Supplemental Figure 2, B and C). These findings suggest that SCF^FBXW11^ specifically mediates the degradation of neurofibromin isoform 1 (containing D3 iso1/GRD1). Given the established role of Ras activation in the neurodevelopment in *Nf1^+/–^* mice and the finding that lower expression levels of isoform 1 are associated with NF1-associated learning deficits, we focused our subsequent efforts on FBXW11 (11, 14, 15, 39).

To assess the direct physical interaction between FBXW11 and GRD1 in live cells, we used a Nanoluciferase-based complementation assay (NanoLuc Binary Technology [BiT]) (40) and plasmids encoding GRD1, GRD2, or FBXW11. This approach splits NanoLuc into small and large BiT fragments (SmBiT and LgBiT) that may be fused to the N- or C-terminus of 2 proteins to test for interaction. Following optimization of the BiT tag orientation on the F-box proteins and neurofibromin GRD1 and GRD2 (Supplemental Figure 2D), interaction between the 2 proteins was detected by complementation of the split luciferase component tags and bioluminescence signal reading in live cells. A significant increase of FBXW11 and GRD1 interaction was detected compared with the control, which was augmented by treatment with the proteasome inhibitor bortezomib (41) (Figure 3C). Consistent with our prior findings, a stronger interaction was observed by NanoBiT complementation assay between FBXO3 and GRD2 as compared with GRD1 (Supplemental Figure 2E). Collectively, these data suggest that FBXW11 binding to the GRD1 domain of neurofibromin induced degradation by the UPP and corroborated our co-IP data, reinforcing preferential interaction between FBXW11 and the neurofibromin GRD1 compared with GRD2.

We next performed in vitro ubiquitination assays to determine whether a direct SCF E3 ubiquitin ligase-substrate interaction existed between FBXW11 and neurofibromin. The ligase activity of an E1, E2 (UbcH3) (42), purified SCF-FBXW11 complex (Supplemental Figure 2F) was assessed using neurofibromin D3 iso1/GRD1 as a peptide substrate. We observed a GRD1-associated ubiquitin smear that was dependent on SCF^FBXW11^ levels and was not detectable in the absence of FBXW11 or GRD1 (Figure 3D). We next used the tandem ubiquitin-binding entities (TUBEs) assay (43) to specifically capture endogenous ubiquitinated neurofibromin in HEK293T cells transfected with either control or FBXW11-targeting siRNA. Western blot analysis following affinity purification with TUBEs-agarose revealed a substantial decrease in ubiquitin-conjugated neurofibromin in cells treated with *FBXW11* siRNA compared with the control (Figure 3E). These results provided additional evidence that the SCF^FBXW11^ complex could mediate the ubiquitination and subsequent proteasomal degradation of neurofibromin.

### A conserved phosphodegron mediates the FBXW11-neurofibromin interaction.

**Figure 4:**
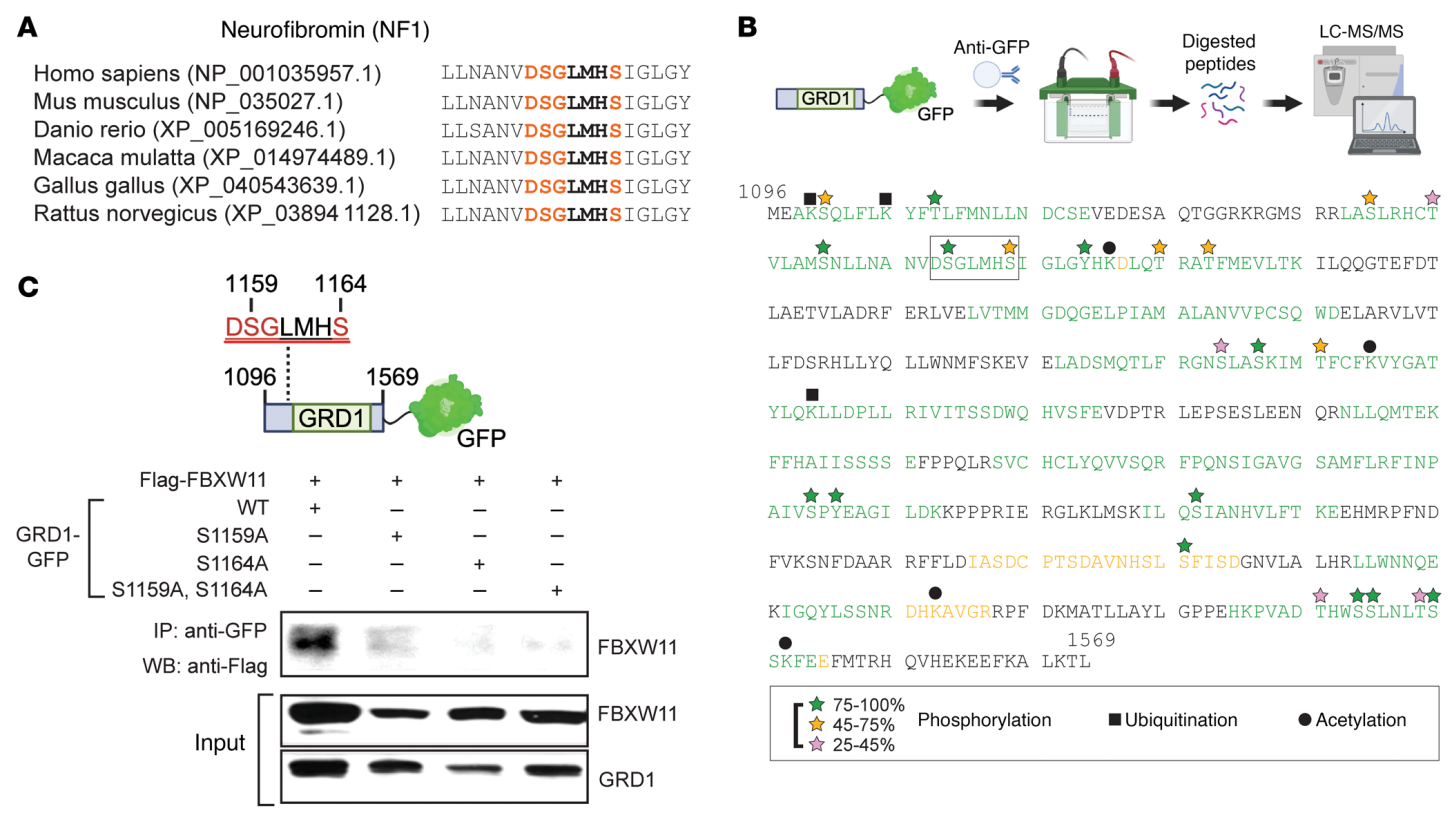
A conserved phosphodegron proximal to the GRD of NF1 isoform 1 is required for interaction with FBXW11. (**A**) NF1 contains a consensus FBXW11 DSGxxxS binding motif near the GRD. (**B**) Summary of posttranslational modifications in NF1-GRD1 detected by LC-MS/MS following affinity purification with an anti-GFP antibody, gel electrophoresis, isolation of the relevant band, and tryptic digestion. Image created in BioRender. Angus, S. (2025) https://BioRender.com/d82r727 (**C**) The indicated mutations were introduced into the putative phosphodegron of NF1-GRD1. The GFP-tagged proteins were cotransfected with Flag-FBXW11 into HEK293T cells. Anti-GFP IP was performed 72 hours after transfection, and FBXW11 was detected by Flag immunoblotting. FBXW11 and GRD1 were detected by immunoblotting from the input (using Flag and GFP antibodies, respectively). Image created in BioRender. Angus, S. (2025) https://BioRender.com/r38z227

Examination of the D3 iso1/GRD1 amino acid sequence revealed a FBXW11 consensus DSGxx(x)S phosphodegron motif (44, 45), DSGLMHS (amino acids 1158–1164 in human neurofibromin isoform 1), which is conserved across multiple species (Figure 4A). To identify potential posttranslational modifications of this domain, we conducted mass spectrometry analysis using gel-purified GRD1 from HEK293T cells. This analysis revealed endogenous phosphorylation of serine 1159 (S1159) and serine 1164 (S1164) in the candidate GRD1 phosphodegron (Figure 4B). To determine whether the putative GRD1 phosphodegron motif regulated binding to FBXW11, we used site-directed mutagenesis to convert the identified serine residues to alanine (A) within the D3 iso1/GRD1. By co-IP assay, we found that mutation of 1 or both conserved serine residues (S1159A, S1164A, and S1159A/S1164A) substantially reduced the observed interaction between FBXW11 and D3 iso1/GRD1 of neurofibromin (Figure 4C). These results suggest that the serine residues of the phosphodegron motif (DSGLMHS) in neurofibromin are critical for its interaction with FBXW11 and substantiate a role for SCF^FBXW11^ in the regulation of neurofibromin protein levels.

### Fbxw11 haploinsufficiency rescues the neurodevelopmental deficits exhibited by Nf1^+/–^ mice.

**Figure 5:**
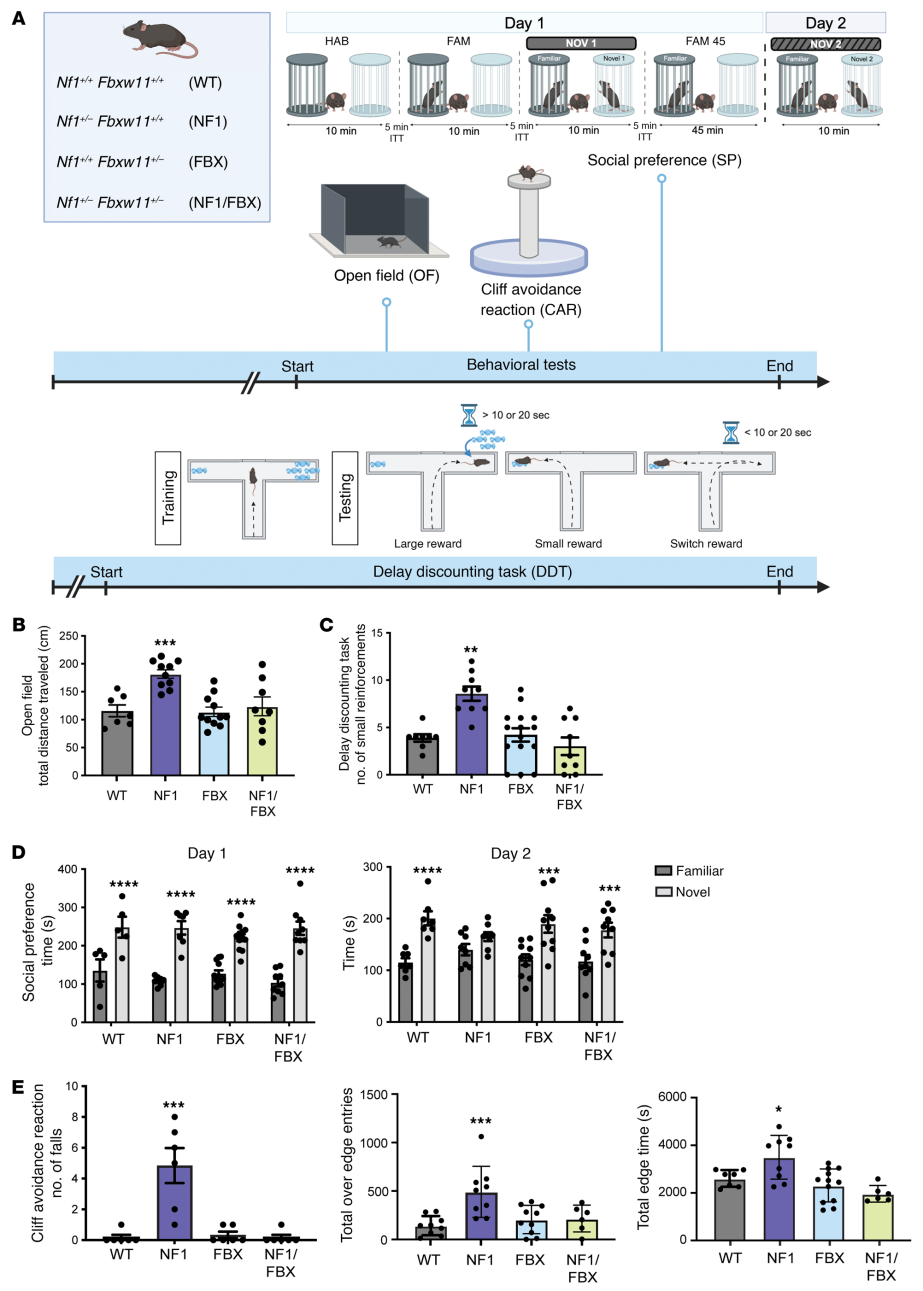
Germline *Fbxw11* knockdown in male *Nf1^+/–^* mice attenuates cognitive deficits. (**A**) The 4 genotypes of genetically engineered mice evaluated are shown. One group (*n* = 8 per genotype) performed the OF, CAR, and SP tasks. An independent cohort (*n* = 9 per genotype) performed the DDT due to the time-intensive training period. The indicated genotypes are abbreviated in parentheses for **B**–**E**. Image created in BioRender. Angus, S. (2025) https://BioRender.com/g70k540 (**B**) *Nf1^+/–^ Fbxw11^+/+^* male mice exhibited hyperactive behavior in the OF test, with increased distance traveled over the 1-hour time period compared with WT *Nf1^+/+^ Fbxw11^+/+^* mice (*P* = 0.0007, by 1-way ANOVA with Dunnett’s multiple-comparison test; mean ± SEM). For heterozygous *Nf1* mutant mice (*Nf1^+/–^ Fbxw11^+/+^*), *Fbxw11* knockdown reduced hyperactive behavior to levels not significantly different from WT control mice. *Fbxw11* had no adverse effects in WT *Nf1^+/+^ Fbxw11^+/–^*. Similar patterns of behavioral rescue were demonstrated in the DDT (**C**), SP (**D**), and CAR (**E**) tasks. (**C**) For the DDT, *Nf1^+/–^ Fbxw11^+/+^* mice showed increased small impulsive reward choices (including small and switch choices) rather than large delayed reward choices compared with WT animals (*P* = 0.0017, by 1-way ANOVA with Dunnett’s multiple-comparison test, mean ± SEM), and *Nf1^+/+^ Fbxw11^+/–^* mice showed no adverse behavioral effects. (**D**) In the SP task, we expected increased time spent with the novel mouse compared with the familiar mouse on day 1 and day 2 for the *Nf1^+/+^ Fbxw11^+/+^* and *Nf1^+/+^ Fbxw11^+/–^* control groups. *Nf1^+/–^ Fbxw11^+/+^* mice showed decreased distinction between a novel partner mouse and a familiar partner mouse on day 2 (*P* = 0.1807), while *Nf1^+/–^ Fbxw11^+/–^* mice showed a preference for the novel partner mouse on day 1 (*P* < 0.0001) and day 2 (*P* = 0.0010) by mixed-effects analysis and Tukey’s multiple-comparison test (mean ± SEM). (**E**) Although no differences by genotype were identified for edge entries (*P* = 0.0508), the number of falls (*P* < 0.0001), over-edge entries (*P* = 0.0006), and edge time (*P* = 0.0323) were increased for *Nf1^+/–^ Fbxw11^+/+^* mice compared with *Nf1^+/+^ Fbxw11^+/+^*, *Nf1^+/+^ Fbxw11^+/–^*, and *Nf1^+/–^ Fbxw11^+/–^* animals (1-way ANOVA with Dunnett’s multiple-comparison test, mean ± SEM). *P < 0.05, **P < 0.01, ***P < 0.001, and ****P < 0.0001.

To test whether disruption of *Fbxw11* could restore neurofibromin levels and ameliorate the neurodevelopmental phenotypes associated with *Nf1* haploinsufficiency, we generated a genetically engineered mouse model (GEMM) deficient in *Fbxw11*. As prior studies in a related model have shown that germline inactivation of *Fbxw11* (b*-TrCP2*) results in embryonic lethality (46), we used a compound heterozygous model of *Fbxw11* and *Nf1^+/–^* (*Nf1^+/–^ Fbxw11^+/–^*) on an inbred C57BL6J strain background to assess performance on established neurocognitive tasks in male *Nf1^+/–^* mice (11, 14, 15) (Figure 5A). The social preference (SP) task measures autism spectrum disorder–like (ASD-like) social deficits, whereas the open field (OF), cliff avoidance reaction (CAR), and delay discounting task (DDT) experiments assess ADHD-like phenotypes. As expected, *Nf1^+/–^* mice that were WT for *Fbxw11* (NF1) showed increased hyperactivity in the OF task measured by total distance traveled (Figure 5B), chose a higher percentage of small rewards rather than waiting to receive the large reward (Figure 5C), demonstrated a decreased preference for a novel versus a familiar partner mouse on day 2 of the SP task (Figure 5D), and had an increase in the number of falls, over-the-edge time, and edge time while performing the CAR task (Figure 5E). *Fbxw11* haploinsufficiency rescued all of these behavioral deficits to the extent that no statistical differences were observed between control WT and *Nf1^+/–^ Fbxw11^+/–^* mice (NF1/FBX) in the performance of these tasks (Figure 5, B–E). Importantly, *Nf1^+/+^ Fbxw11^+/–^* mice (FBX) were phenotypically normal and did not exhibit neurodevelopmental abnormalities.

**Figure 6:**
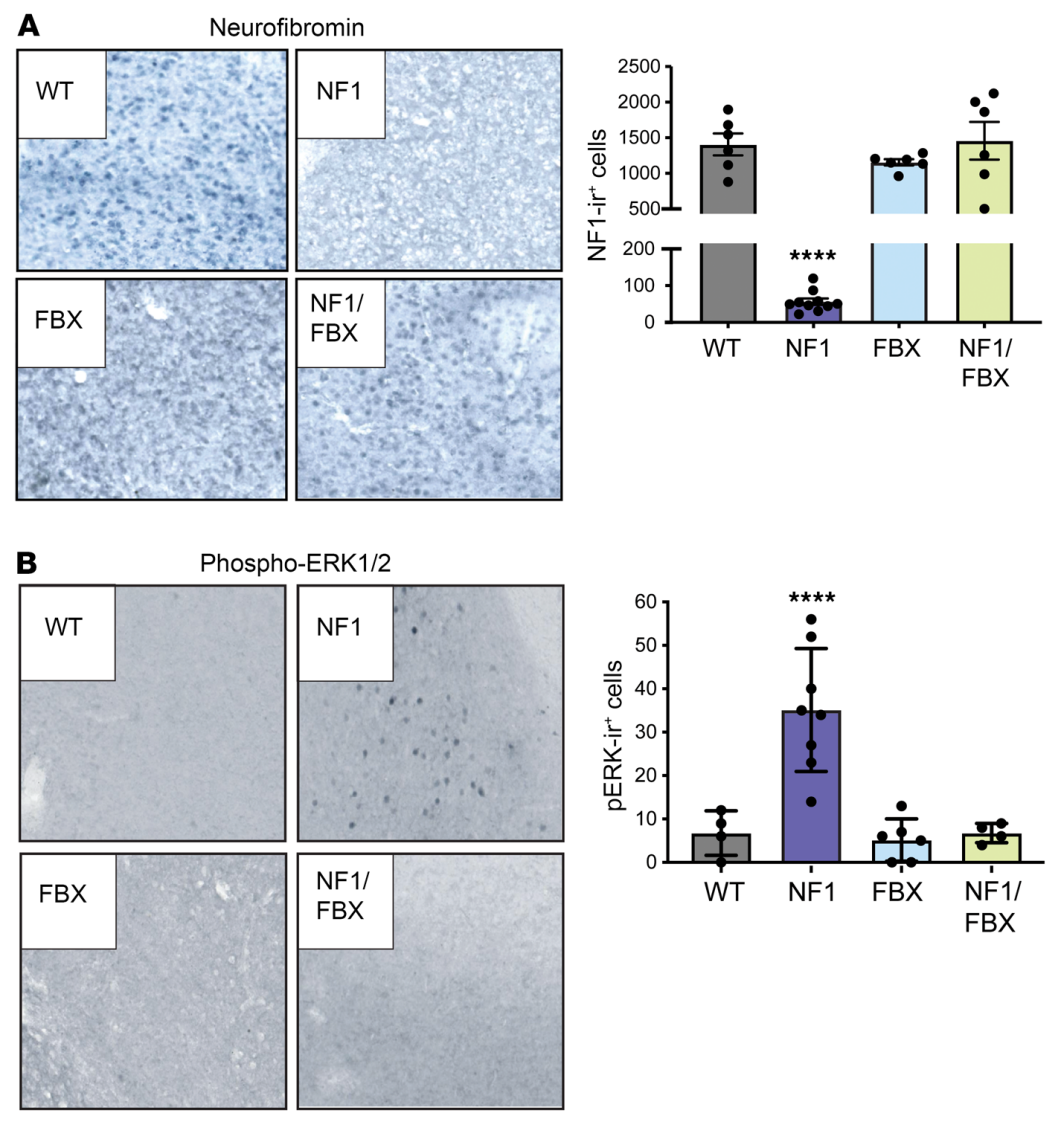
Germline *Fbxw11* knockdown restores neurofibromin expression and suppresses ERK hyperactivation in *Nf1^+/–^* murine brain tissue. IHC staining for (**A**) neurofibromin and (**B**) pERK1/2 in brain tissue from mice from Figure 5 was performed to determine the effect of heterozygous knockdown of *Fbxw11*, with representative images and quantification of immunoreactive cells shown. Original magnification, ×200. ****P < 0.0001, by 1-way ANOVA with Tukey’s multiple-comparison test. Data indicate the mean ± SEM.

Correlative IHC analyses showed that neurofibromin levels were significantly increased (Figure 6A) and phosphorylated ERK1/2 (pERK1/2) levels were reduced (Figure 6B) in brain tissue from *Nf1^+/–^ Fbxw11^+/–^* mice compared with that from *Nf1^+/–^* mice. Collectively, these results indicate that germline *Fbxw11* haploinsufficiency increased neurofibromin levels, attenuated ERK activation, and rescued the neurodevelopmental deficits exhibited by *Nf1^+/–^* mice.

### Targeted Fbxw11 ablation in the nucleus accumbens corrects Nf1-related neurobehavioral deficits.

**Figure 7:**
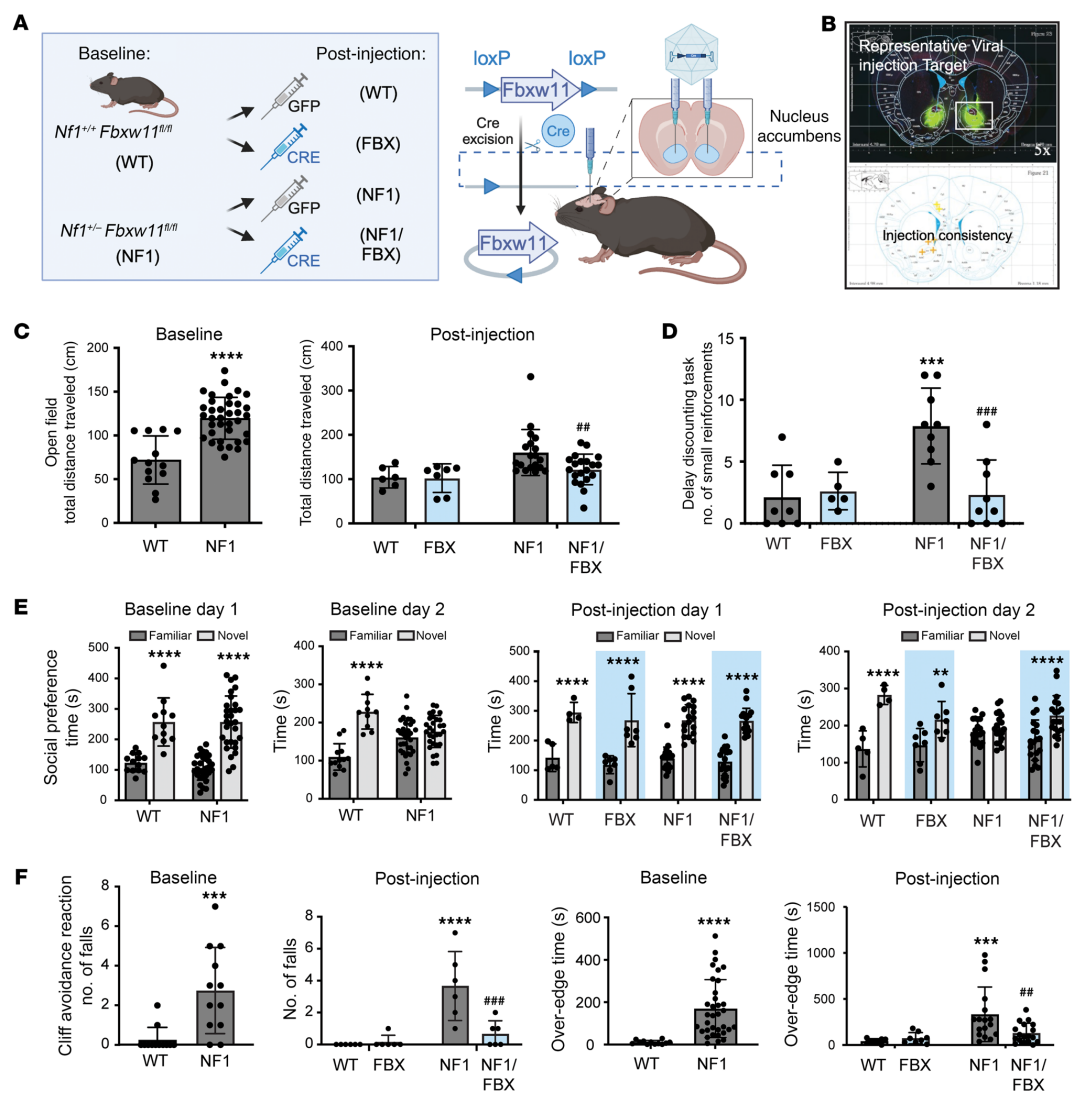
Selective deletion of *Fbxw11* in the nucleus accumbens rescues cognitive deficits in male *Nf1^+/–^ Fbxw11^fl/fl^* mice. (**A**) Schematic of stereotaxic AAV virus injection. Cohorts of male *Nf1^+/–^ Fbxw11^fl/fl^* and control *Nf1^+/+^ Fbxw11^fl/fl^* mice received bilateral injections of AAV-GFP (control, *n* = 8 mice per genotype) or AAV-Cre-GFP virus (*n* = 18 mice per genotype) for experiments (after injection). Mice underwent behavioral testing at baseline to confirm the expected deficits. The indicated genotypes and treatments are abbreviated as shown in parentheses for group labeling in **C**–**F**. Image created in BioRender. Angus, S. (2025) https://BioRender.com/r74c640 (**B**) To ensure correct viral injection, IHC was performed to visualize GFP expression at the center of each injection site. A representative image of the injection site at the nucleus accumbens and center points of all injections is shown. (**C**–**F**) Studies were performed as in Figure 4. (**C**) *Nf1^+/–^ Fbxw11^fl/fl^* male mice exhibited hyperactivity in the OF task (*P* < 0.0001) at baseline and following GFP injection (*P* = 0.0053) compared with WT mice that was reduced in mice receiving CRE (*P* = 0.0056) as determined by 1-way ANOVA with Dunnett’s multiple-comparison test (mean ± SEM). (**D**) *Nf1^+/–^ Fbxw11^fl/fl^* male mice infected with GFP made more impulsive choices in the DDT when compared with WT mice receiving GFP (*P* = 0.0001) that was suppressed in the CRE group. *P* = 0.0001, by 1-way ANOVA with Dunnett’s multiple-comparison test. Data indicate the mean ± SEM. (**E**) *Nf1^+/–^ Fbxw11^fl/fl^* male mice spent more time investigating a novel partner on day 1 (*P* < 0.0001) but not day 2 (*P* = 0.1625) at baseline in the SP task. This pattern persisted in *Nf1^+/–^ Fbxw11^fl/fl^* male mice receiving GFP, while CRE led to increased interaction time with a novel mouse versus a familiar mouse on day 2 (*P* < 0.0001). Significance was determined by mixed-effects analysis and Tukey’s multiple-comparison test. Data indicate the mean ± SEM. (**F**) *Nf1^+/–^ Fbxw11^fl/fl^* male mice had a greater number of falls and over-the-edge time at baseline (*P* = 0.0009 and *P* < 0.0001, respectively) and after injection with GFP (*P* < 0.0001 and *P* = 0.0007, respectively) compared with WT mice in the CAR task. After CRE injection, the number of falls (*P* = 0.0003) and over-edge time (*P* = 0.002) for *Nf1^+/–^ Fbxw11^fl/fl^* male mice was reduced compared with the GFP-injected cohort (NF1/FBX vs. NF1). Significance was determined by 1-way ANOVA with Dunnett’s multiple-comparison test. Data indicate the mean ± SEM. **P < 0.01, ***P < 0.001, and ****P < 0.0001. ^##^*P* < 0.01 and ^###^*P* < 0.001, for comparison of CRE-mediated *Fbxw11* ablation with control GFP (NF1/FBX to NF1).

The nucleus accumbens (NAc) is a critical component of the ventral striatum, and it plays a pivotal role in the striatal circuitry by integrating signals related to reward, motivation, pleasure, and reinforcement learning (47, 48). Prior work suggests a role for NAc dysfunction in the manifestations of both ASD and ADHD, as well as in neurocognitive deficits associated with NF1 (47, 49–53). As such, we next investigated whether targeted CNS ablation of *Fbxw11* gene function within the NAc could also rescue the neurocognitive deficits exhibited by *Nf1^+/–^* mice. For these studies, we generated *Nf1^+/–^ Fbxw11^fl/fl^* mice, which enabled adeno-associated virus 5–mediated (AAV5-mediated) delivery of Cre-recombinase fused to GFP (CRE) to conditionally inactivate *Fbxw11* (Figure 7A). Control AAV5-GFP (GFP) or AAV5-Cre-GFP (CRE) virus was injected bilaterally into the NAc, and mouse performance was assessed on the previously described neurocognitive tasks (Figure 7B). Prior to injection, all animals exhibited the expected baseline performance deficits on the indicated tasks, with no statistical differences seen between animals that were randomly assigned to receive GFP or CRE injection (SP: *F*_(1, 40)_ = 0.01156, *P* = 0.9149; OF: *F*(_1, 45)_ = 1.334, *P* = 0.2542; CAR: F_(1, 48)_ = 0.4834, *P* = 0.4902) (Figure 7, C–F). Strikingly, in the OF task, CRE-injected *Nf1^+/–^ Fbxw11^fl/fl^* mice (NF1/FBX) exhibited reduced hyperactivity with decreased total distance traveled, similar to WT mice (Figure 7C). In the DDT test, NF1/FBX mice exhibited large delayed choices over small impulsive choices after CRE injection (Figure 7D). In the SP task, NF1/FBX mice showed restored preference for a novel partner over a familiar partner on day 2 (Figure 7E). Finally, in the CAR task, which measures risk-taking behavior, NF1/FBX mice exhibited reduced edge and over-the-edge times (Figure 7F). Collectively, these findings revealed that NAc-targeted disruption of *Fbxw11* was sufficient to rescue the neurobehavioral phenotypic deficits exhibited by *Nf1^+/–^* male mice.

**Figure 8:**
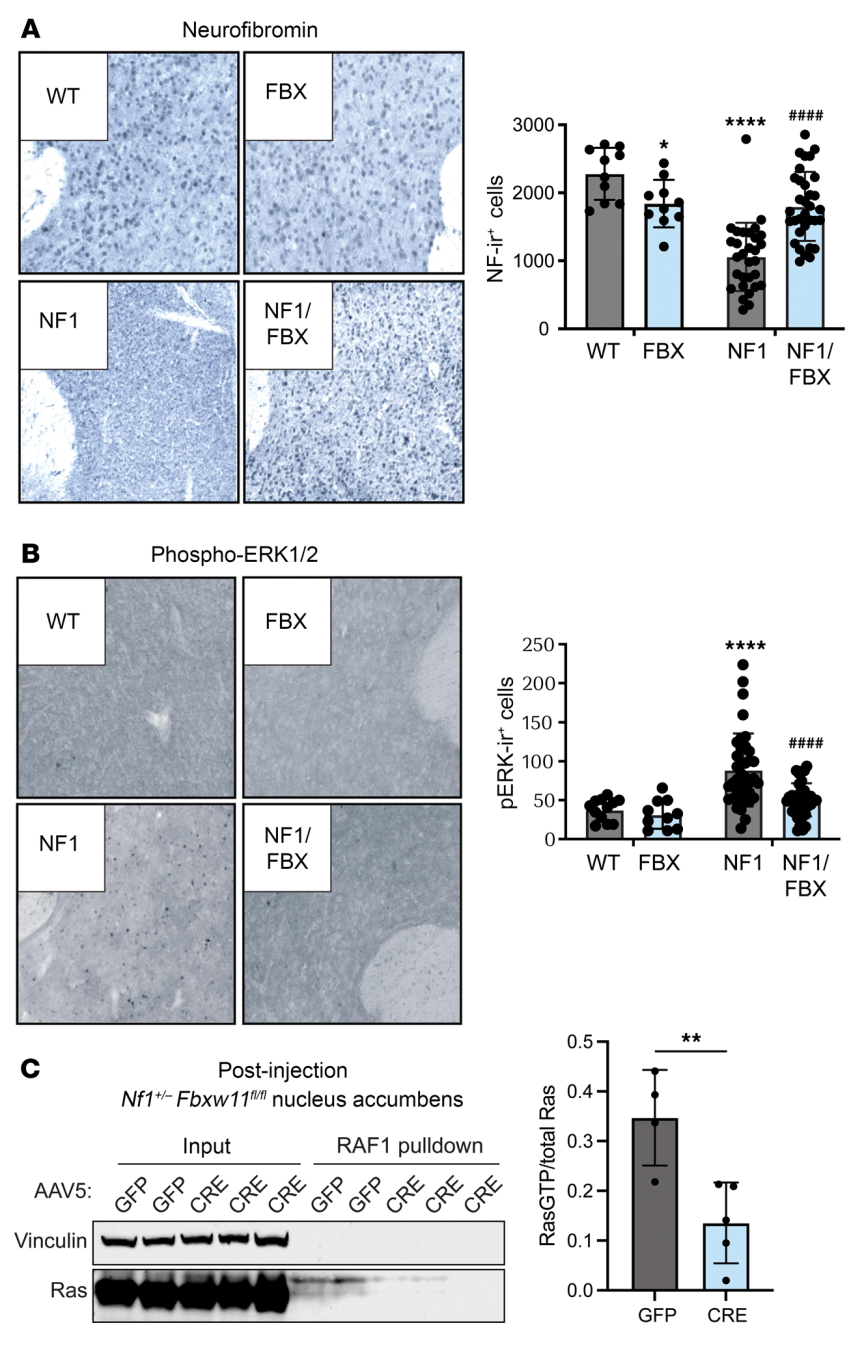
Targeted deletion of Fbxw11 in the nucleus accumbens of *Nf1^+/–^* mice corrects Nf1/Ras/MAPK pathway activity. (**A** and **B**) IHC staining for neurofibromin (**A**) and pERK1/2 (**B**) was performed to determine the effect of targeted ablation of *Fbxw11*, with representative images and quantification of immunoreactive cells from mice as indicated in Figure 7. Original magnification, ×200. (**C**) Brain tissue from the nucleus accumbens region of mice of the indicated genotype and injected with the indicated AAV was used to generate lysate for RAF1 pull-down assays. Immunoblotting was used to detect vinculin (loading control) and Ras. *P < 0.05, **P < 0.01, and ****P < 0.0001. ^####^*P* < 0.0001, for comparison of CRE-mediated *Fbxw11* ablation with control GFP (NF1/FBX to NF1). One-way ANOVA with Tukey’s multiple-comparison test. Data indicate the mean ± SEM.

To confirm that the changes in neurobehavioral phenotypes were associated with increased neurofibromin and an effect on Ras pathway signaling, the injected regions of brain tissue were subsequently evaluated. IHC analysis of brain tissues confirmed increased neurofibromin levels (Figure 8A) and a reduction of pERK1/2 (Figure 8B). As further confirmation of neurofibromin function, protein was extracted from brain tissue of *Nf1^+/–^ Fbxw11^fl/fl^* mice from these studies that had been injected with GFP or CRE AAV. Lysates were used for IP experiments with a Ras-binding domain (RBD) Raf peptide to capture active, GTP-bound Ras. Consistent with our expectation, immunoblotting for bound Ras confirmed that active Ras-GTP levels were reduced in the injected areas of the murine brain following CRE injection when compared with the GFP control (Figure 8C). Collectively, these data demonstrate that targeted disruption of *Fbxw11* in a functionally relevant region of the adult mouse brain increased neurofibromin protein levels, suppressed hyperactive Ras signaling, and rescued neurodevelopmental deficits in haploinsufficient male *Nf1^+/–^* animals.

## Discussion

Reduced levels of essential proteins resulting from haploinsufficiency are known to affect over 600 diseases, including a range of metabolic, neurodegenerative, and neurodevelopmental conditions, such as those affecting individuals with NF1 (54–56). While the precise mechanisms underlying the neurological complications experienced by patients with NF1 remain incompletely understood, it is likely that disruption of both Ras-dependent and -independent processes occurring secondary to loss of neurofibromin plays a role (57–61). Given the biological function of neurofibromin as a GTPase for Ras, a key oncogene integral to 30% of all malignancies, repurposing therapeutics that target specific downstream Ras pathways is one consideration. An alternative approach involves restoring endogenous neurofibromin protein levels to a physiologically sufficient range by stabilizing and preventing the degradation of the residual protein produced by the functional allele (23, 24). Here, we demonstrate that suppression of FBXW11-mediated degradation of neurofibromin ameliorated neurodevelopmental phenotypes and diminished the Ras hyperactivation seen in preclinical genetic mouse models of NF1. These findings warrant ongoing preclinical studies evaluating this therapeutic strategy for the treatment of haploinsufficient NF1 disease manifestations. Furthermore, this strategy could be applied to identify interventional approaches targeting specific UPP factors for other pathologic conditions driven by haploinsufficiency.

Prior data suggest that, given the differences in Ras-GAP potency and tissue distribution, isoform 1 (D3 iso1/GRD1) and isoform 2 (D3 iso2/GRD2) likely possess distinct functional repertoires (35, 62). While both F-box proteins targeted the GRD of neurofibromin, we show that FBXW11 preferentially bound isoform 1 (D3 iso1/GRD1), whereas FBXO3 exhibits specificity for isoform 2 (D3 iso2/GRD2) (35, 36, 38). Importantly, decreased levels of isoform 1 are associated with the occurrence of learning disabilities and neurocognitive deficits in patients with NF1 (39), whereas isoform 2 is known to be critical for cardiovascular development (62, 63). For these reasons, we focused our attention on the effect of suppressing FBXW11-mediated degradation of neurofibromin isoform 1. In patients with NF1, ADHD and ASD are common nonmalignant manifestations. In mice, these phenotypes can be readily assessed by measuring performance on a battery of specific tasks that evaluate hyperactivity and impulsivity (e.g., OF, CAR, and DDT) and social behavior (e.g., SP). Strikingly, we found that germline heterozygous deletion of *Fbxw11* (*Nf1^+/–^ Fbxw11^+/–^*) partially modulated the degradation of neurofibromin and was able to rescue the *Nf1* haploinsufficient phenotype. This observation provides critical insight and suggests that therapeutic latitude exists for FBXW11 that does not require its complete ablation for phenotypic rescue.

In complementary experiments, we evaluated the effect of targeted disruption of *Fbxw11* within the NAc in adult *Nf1^+/–^* mice. The NAc plays an important role in modulating cognitive control and reward valuation in ADHD and NF1 (15, 49, 64–66). Dysfunction of the NAc has been proposed to underlie the neurocognitive manifestations of ASD, ADHD, and NF1 (47, 49–53). Specifically, in animal models and patients with ADHD, PET imaging reveals an association between lower motivation and synaptic dopamine markers within the NAc (67). In addition, functional neuroimaging studies indicate that response inhibition relies on reward circuitry brain areas including the NAc, which shows reduced signals in clinical samples from patients with NF1 (53, 68, 69). Accordingly, preclinical studies using murine experimental systems demonstrate that lesions of the NAc increase impulsivity on the DDT (70–73). Last, the NAc plays a role in driving motivated social interactions, and its dysfunction can result in reduced motivation, impaired reward processing, and emotional difficulties leading to social withdrawal or inappropriate social behavior (74, 75). Consistent with these findings, we show that targeted disruption of *Fbxw11* within the NAc in adult mice rescued performance on all experimental tasks performed to assess hyperactivity/impulsivity and social behavior. Importantly, this observation reveals that correction of neurological deficits was not restricted to a developmental window and suggests potential efficacy for targeted therapeutic approaches well beyond the early stages of neurodevelopment.

The rescue of neurodevelopmental phenotypes by *Fbxw11* suppression in both models was associated with immunohistochemical and biochemical evidence of increased expression of neurofibromin and subsequent decreased Ras/MAPK pathway activation within the brain. No adverse effects were appreciated in mice with germline heterozygous loss of *Fbxw11*, nor in those having undergone targeted *Fbxw11* inhibition within the NAc. We did not perform tissue- or cell-type–specific analyses beyond our investigation of the CNS and the NAc. While no gross abnormalities were observed and the mice developed normally and had a normal lifespan, it will be important to study potential cell-type and tissue-type dependencies of FBXW11 substrates in a dose-dependent fashion. Furthermore, although other previously identified UPP complexes could contribute to neurofibromin degradation (19–21), our genetic models establish the phenotypic rescue capacity of FBXW11 specifically.

In terms of future drug development, our data demonstrate a dose-dependent role of FBXW11 in neurofibromin stability. However, we acknowledge that there are over 30 known substrates of FBXW11, including β-catenin, IkB, p19Arf, and IL-17 receptor A (46, 76, 77). Each affected substrate or pathway could have a significant consequence in response to FBXW11-targeted small molecules and should be evaluated in therapeutic development. In 7 individuals, Holt et al. identified variants that were predicted to destabilize the respective peptides encoded by the FBXW11 gene that were associated with developmental abnormalities of the eye and digits, and effects on neurodevelopment (78). These findings underscore the importance of considering the effect of FBXW11 inhibition in humans, especially during early development. Functionally, the development of therapeutically useful drugs that modulate F-box proteins may be a challenge (79). However, newer technologies such as proteolysis-targeting chimeras (PROTACs) would be one potential approach to pharmacologically target FBXW11. Additionally, the identification of the phosphodegron in neurofibromin raises the potential for identifying kinases that modulate the binding between neurofibromin and the E3 ligase complex (Figure 3), allowing for the repurposing of existing kinase inhibitors to stabilize neurofibromin. Again, the systemic effects of kinase inhibition need to be explored to limit toxicity. Both therapeutic approaches are the subject of ongoing studies in our laboratory.

It is important to consider the phenotypic variability of ADHD and ASD based on our findings. *Nf1^+/–^* mice most closely resemble the genetic features of patients with nonsense or frameshift mutations leading to a premature termination codon in *NF1* that disrupts Ras-GAP activity through the formation of a truncated protein. Clinically, these nonsense and frameshift variants are generally associated with increased disease severity (80). Intriguingly, certain missense mutations, even those outside the Ras-GAP domain, have been associated with cognitive deficits and increased risk for tumorigenesis. Emerging data demonstrate that specific neurofibromin variants can function in a dominant negative fashion, accelerating degradation of the WT protein via dimerization (81). The effect of FBXW11 depletion on neurofibromin stabilization in the context of these missense variants warrants further investigation. In the *Nf1^+/–^* murine model used in our studies, the null allele contained an in-frame insertion predicted to produce a larger protein due to inclusion of a portion of the *neo* transgene (82). However, the predicted protein was never observed in the original publication or in our own studies. Analysis of behavioral phenotypes (and the extent of rescue by a UPP/FBXW11 targeting strategy) in other clinically relevant murine models of NF1, both missense and nonsense, will be critical. In summary, the frequency and severity of neurodevelopmental sequelae in children with NF1 highlight a great need for therapeutic improvement. While some studies have shown varying degrees of improvement in learning and memory with short-term MEK inhibition, the extended blockade of this pathway during critical periods of brain development may interfere with neuronal plasticity, synaptic development, and neurogenesis (83–85). This is particularly relevant in children, in whom long-term MEK inhibition could have unintended consequences related to normal brain maturation. Additionally, MEK inhibition can be associated with notable adverse effects, leading to dose reductions or discontinuation of therapy, thereby limiting its long-term use for the treatment of NF1-associated ADHD and ASD (86). Consequently, the identification of specific targets and therapeutic strategies to ameliorate NF1-associated neurocognitive deficits through restored neurofibromin function is of paramount importance. In this study, we have identified FBXW11 as a critical regulator of neurofibromin degradation and show that its inhibition rescues the *Nf1* haploinsufficient phenotype. Our findings indicate that stabilization of neurofibromin is a promising therapeutic strategy that warrants future evaluation for the treatment of haploinsufficient disease manifestations of NF1.

## Methods

### Sex as a biological variable

Male *Nf1^+/–^* mice were utilized for these studies due to their well-documented neurobehavioral phenotype (15). While our findings are likely applicable to female mice as well, recent evidence suggests sexual dimorphism in *Nf1^+/–^* mice, and alternative assays may need to be performed in future studies (87).

### Animals

*Nf1* heterozygous (*Nf1^+/–^*, C57BL/6) mice have been previously described (82, 88). *Fbxw11* heterozygous (*Fbxw11^+/–^*, C57BL/6NTac) mice were from Knockout Mouse Project (KOMP) (strain ID: *Fbxw11^tm1a(KOMP)Wtsi^*, design ID: 49846, project ID: CSD25672, MGI ID: 4363386). *Fbxw11^fl/fl^* (exon 4 targeted) mice were generated from the *Fbxw11^tm1a(KOMP)Wtsi^* strain by FLP-FRT recombination and subsequently crossed with *Nf1^+/–^* mice.

### Genotyping

*Nf1* genotyping was performed as previously described (88). *Fbxw11* was genotyped by PCR using forward primer F (5′-GAATCTGTGTTACCAGGCACTCAGC-3′) and reverse primer ttR (5′-GCCTTAGCTCACTATTCCCCATTGC-3′) for WT (631 bp) and primer neoF (5′-GGGATCTCATGCTGGAGTTCTTCG-3′) as the reverse primer ttR for KO (633 bp). The annealing temperatures were 56°C and 59°C, respectively. For *Fbxw11^fl/fl^* genotyping, primer F and reverse primer ttR were used (826 bp).

### Cell culture

HEK293T cells (American Type Culture Collection [ATCC], CRL-3216), HeLa cells (ATCC CCL-2), MEFs (generated in our laboratory), and human fibroblasts (ATCC, PCS-201-012) were grown in DMEM containing 10%–12% FBS (Atlanta Biologics) and penicillin/streptomycin solution (1:100; MilliporeSigma). LUVA cells (Kerafast) were cultured in STEM PRO-34 SFM (10640, Gibco, Thermo Fisher Scientific), STEM PRO-34 nutrient supplement (10641-025, Gibco, Thermo Fisher Scientific), penicillin/streptomycin solution, and 2 mM l-glutamine (25030-081, Gibco, Thermo Fisher Scientific) (89).

### Chemical reagents, siRNAs, cDNAs and recombinant proteins

The following chemical reagents, siRNAs, cDNAS, and recombinant proteins were used. Synthesized ANVDSGLMHSIGLGYHK peptides (Selleckchem), MG132 (Tocris), bortezomib (Selleckchem), CHX (MilliporeSigma), BC-1215 (MilliporeSigma), pyrrolidinedithiocarbamate ammonium (PDTC) (P8765, MilliporeSigma), siPORT NeoFX Transfection Reagent (Ambion), ECL Western blotting detection reagent (Amersham Biosciences), IPP: 200 units/mg (catalog 10108987001, Roche), E2: His-UbcH3/Cdc34, human recombinant (catalog E2-610, BostonBiochem); Flag peptide (3290, MilliporeSigma), and polyethylenimine (PEI) 25 kDa linear (catalog 23966-2, Polysciences); human F-box siRNA library (Ambion), human FBXW11 siRNA (siRNA ID 23487, Ambion), human FBXO3 siRNA (siRNA ID 25346, Ambion), human FBXW11-3 unique 27 mer siRNA duplexes (SR308161, Origene), human FBXW1/b-TrCP1 siRNA (SASI_Hs01_00189438 and SASI_Hs01_00189439, MilliporeSigma), and Mission siRNA universal negative control 1 (SIC002, MilliporeSigma); GFP-tagged human neurofibromin, transcript variant 1 cDNA (RG220425, Origene), Myc-DDK–tagged human FBXO3 transcript variant 1 cDNA (RC208494, Origene), Myc-DDK–tagged human FBXW11 transcript variant 3 cDNA (RC218905, Origene), Myc-DDK–tagged human FBXW7 transcript variant 1 cDNA (RC217398, Origene), human RBX1 cDNA (SC115112, Origene), human SKP1 transcript variant 1 cDNA (SC126980, Origene), and human CUL1 cDNA (SC108409, Origene); human FBXW11 recombinant protein (H00023291-P01, Abnova), human CUL1 recombinant protein (H00008454-P01, Abnova), human RBX1 recombinant protein (H00009978-P01, Abnova), and human SKP1 recombinant protein (H00006500-P01, Abnova).

### siRNA screening and siRNA transfection

Cell suspensions were incubated in complete medium (without antibiotics) at 37°C in polypropylene tubes for less than 1 hour until used in the chemical reverse transfection. Transfection complexes were prepared in Opti-MEM serum-free medium (Invitrogen, Thermo Fisher Scientific) by mixing 0.3 μL siPORT NeoFX Transfection Reagent (Ambion, Thermo Fisher Scientific) and 10 nM siRNA (individual siRNA members of the F-boxes, Ambion) (90). Human fibroblasts (2 × 10^5^ cells/well) and LUVA (5 × 10^5^ cells/well) were plated in a 6-well format simultaneously with addition of transfection complexes. Cells were incubated for 72 hours and subsequently analyzed by Western blotting. Confirmation was performed with a second siRNA targeting FBXW11 or FBXO3 using 3 siRNA constructs and scrambled siRNA in HeLa or HEK293T cells. The transfection method was the same as described above. After siRNA constructs were tested for 72 hours of transfection, 1 was chosen for further experimentation.

### CHX chase analysis

Neurofibromin stability was assessed using CHX chase in HeLa cells (91). After 48–72 hours of transfection, cells were treated with 20 μg/mL CHX (MilliporeSigma) for the indicated durations. The cell lysates were prepared and analyzed by Western blotting.

### Construction of GFP (or Flag) fusions with domains of neurofibromin

Full-length human neurofibromin cDNA was purchased from Origene. Neurofibromin gene fragments were synthesized using various 5′ primers and 3′ primers and subcloned into the SgfI/MluI sites of pCMV-AC-GFP (or pCMV6-ENTRY, Origene). The pEGFP-C1 plasmid containing the NF1-GRD iso1 gene fragment (92) was used as a template. Domain D1: forward primer, 5′-GAGGCGATCGCCATGGCCGCGCACAGGCCGGTGGA-3′; reverse primer, 5′-GCGACGCGTATAGTTAAGGATTAGCTTTGTTGC-3′. Domain D2: forward primer, 5′-GAGGCGATCGCCCCAAAAGCCAAAATGGAAGATGG-3′; reverse primer, 5′-GCGACGCGTTGGAGGACCCAGGTATGCAAGAAG-3′. Domain D3iso2/GRD2: forward primer, 5′-GAGGCGATCGCCATGGAAGCCAAATCACAGTTATTTC-3′; reverse primer, 5′-GCGACGCGTTAAGGTTTTCAAAGCCTTGAATTCTTC-3′. Domain D3iso1/GRD1: forward primer, 5′-GAGGCGATCGCCATGGAAGCCAAATCACAGTTATTTC-3′; reverse primer, 5′-GCGACGCGTTAAGGTTTTCAAAGCCTTGAATTCTTC-3′. Domain D4: forward primer, 5′-GAGGCGATCGCCGAGCACAAACCTGTGGCAGATAC-3′; reverse primer, 5′-GCGACGCGTGCCCTGGTTTGCAATGGTTAAGGT-3′. Domain D5: forward primer, 5′-GAGGCGATCGCCACGCCGCTCACCTTCATGCACCA-3′; reverse primer, 5′-GCGACGCGTCACGATCTTCTTAATGCTATTACG-3′.

### cDNA transfection and expression

Transfections were performed using PEI (93) in HEK293T cells, and expression was measured 48–72 hours later by Western blotting. PEI (1 mg/mL in sterile water) was neutralized with HCl and filtered at 0.2 μm (MilliporeSigma), and PEI solution and plasmids were mixed at a 5:1 ratio.

### Co-IP

Cell extracts were prepared in IP lysis buffer of (1% (v/v) Triton X-100, 25 mM HEPES, pH 7.5, 150 mM NaCl, 0.2 mM EDTA, 0.5 mM DTT, and 1 mM PMSF, supplemented with phosphatase and protease inhibitors. Flag-tagged FBXW11 or FBXO3 cDNA plasmids and GFP-tagged truncated NF1 cDNA plasmids were cotransfected into HEK293T cells. Sixty-eight to 72 hours after transfection, HEK293T cells were incubated with MG132 (15 mM) for 6 hours and lysed, sonicated, and centrifuged for 25 minutes at 16,000*g*. Supernatants were incubated with anti-FLAG M2 affinity gel at 4°C overnight. Immunoprecipitated proteins were washed 3 times with 250 mM NaCl/IP lysis buffer and eluted by boiling in Laemmli sample buffer. Samples were analyzed by SDS-PAGE and anti-GFP immunoblotting.

### Western blotting

Whole-cell or tissue extracts were prepared with xTractor lysis buffer (Clontech) including a protease inhibitor (Complete Mini EDTA-free, Roche) and a phosphatase inhibitor (PhosSTOP, Roche). After sonication and centrifugation for 5 minutes at 10,000*g*, the supernatants were collected. Proteins were separated by SDS-PAGE (4%–12% gradient gels), transferred onto PVDF membranes, incubated with a blocking buffer, the primary and appropriate secondary antibodies, and developed with ECL (catalog 32134, Thermo Fisher Scientific). The following primary antibodies were used: neurofibromin/NF1 (sc-67, sc-376886, Santa Cruz Biotechnology and ab238142, Abcam); Flag (F7425, MilliporeSigma); GFP (ab1218, Abcam, Western blotting and Invitrogen, IP); turboGFP (AB513, Evrogen); FBXW11/bTrCP2 (GTX33193, GeneTex and PA5-109715, Invitrogen, Thermo Fisher Scientific); ERK1/2 (9102, Cell Signaling Technology); anti-pERK Thr202/Tyr204 (4377, Cell Signaling Technology); IκBα (9242 Cell Signaling Technology); β-catenin (8480, Cell Signaling Technology); ubiquitin (3933, Cell Signaling Technology); CUL1 (17775, Cell Signaling Technology); SKP1 (2156, Cell Signaling Technology); Rbx1 (4397, Cell Signaling Technology); and GAPDH (2118, Cell Signaling Technology). The following secondary antibodies were used: goat anti–mouse IgG HRP (31430, Invitrogen, Thermo Fisher Scientific) and goat anti–rabbit IgG HRP (31460, Invitrogen, Thermo Fisher Scientific).

### Purification of the SCF-FBXW11 complex

Thirty 150 mm plates of HEK293T cells were transfected using PEI with the indicated constructs (Flag-FBXW11, CUL1, SKP1, and RBX1). Seventy-two hours later, cells were washed twice with PBS, flash-frozen, and lysed in 50 mL IP lysis buffer (1% Triton X-100, 25 mM HEPES, pH7.5, 150 mM NaCl, 0.2 mM EDTA, 2 mM sodium orthovanadate, 2 mM sodium fluoride, 0.5 mM DTT, 1 mM PMSF and proteinase inhibitor) by sonication. Lysates were cleared by centrifugation at 20,000*g* for 20 minutes at 4°C and incubated with 150 μL anti-FLAG M2 affinity gel resin (MilliporeSigma) overnight at 4°C, washed 7 times with 500 μL wash buffer (10 mM Tris, pH = 7.4, 100 mM KCl and 0.5% Nonidet P-40), and eluted with 500 μL elution buffer I (100 μg/mL 3X FLAG peptide [MilliporeSigma], 0.4 μg/mL, 20 mM Tris, pH 8.0, 20% glycerol, 100 mM KCl, and 1 mM DTT) for 30 minutes at 4°C with gentle rocking. The supernatant was collected and stored at –20°C until further analysis.

### Site-directed mutagenesis

Plasmid pEGFP-C1 containing the NF1-GRD gene fragment (92) was used as a mutagenesis template. Plasmids were propagated in TOP10 *E*. *coli* (Invitrogen, Thermo Fisher Scientific) and isolated using Qiagen Miniprep kits (Qiagen). Primers were synthesized by Invitrogen and purified by SePOP desalting. The melting temperature of the DNA duplex was calculated as Tm = 81.5 + 0.41 (percentage GC [guanine (G) and cytosine (C) bases in the DNA sequence]) – (675/N [total number of nucleotides in the DNA sequence]) – percentage mismatch. PCR reactions of 50 μL contained 50–100 ng template, 1 μM primer pair, 200 μM deoxyribonucleotide triphosphate, and 3 units of pfu DNA polymerase were used according to the manufacturers’ protocol for the QuikChange II XL site-directed mutagenesis kit (Agilent Technologies). Mutations were verified by Sanger sequencing. The following mutagenic oligonucleotide primers were used: S1159A-GRD, forward, 5′-GCCAACGTAGACGCTGGTCTCATGCAC-3′; S1159A-GRD, reverse, 5′-GTGCATGAGACCAGCGTCTACGTTGGC-3′; S1164A-GRD, forward, 5′-GGTCTCATGCACGCCATAGGCTTAGG-3′; S1164A-GRD, reverse, 5′-CCTAAGCCTATGGCGTGCATGAGACC-3′; S1159A-S1164A, forward, 5′-GCCAACGTAGACGCTGGTCTCATGCACGCCATAGGCTTAGG-3′; S1159A-S1164A, reverse: 5′-CCTAAGCCTATGGCGTGCATGAGACCAGCGTCTACGTTGGC-3′.

### In vitro ubiquitination assay

The GRD iso1 ubiquitination assay (Enzo Life Sciences) was carried out according to the manufacturer’s instructions with purified SCF^FBXW11^ complex (E3), purified GRD iso1 (substrate), and His-UbcH3/Cdc34 (E2) (42), in vitro. Ubiquitin ligase activity was determined by Western blotting with anti-Ub antibody (3933, Cell Signaling Technology).

### LC-MS/MS and proteomics analysis

HEK293T cells were plated 1 day prior to transfection at 3.0 × 10^6^ cells per 15 cm tissue culture plate and transfected by PEI with GFP-tagged GRD1 plasmid. Forty-eight hours after transfection, cells were treated with 15 μM MG132 for 4–6 hours, washed once in PBS, and lysed in IP lysis buffer. Clarified lysates were immunoprecipitated with anti-GFP antibody overnight at 4°C and collected with protein A/G beads as described above. GFP-GRD fusion proteins were separated by SDS-PAGE utilizing Coomassie blue staining, and gel bands were analyzed by LC-MS/MS. Tryptic peptides from GFP-GRD1 were injected onto the NanoAcquity UPLC column 1.7 μm BEH130 C18 (100 μm × 100 mm) with a NanoAcquity UPLC Trap column (5 μm Symmetry C18, 180 μm × 20 mm). Peptides were eluted with a linear gradient (3%–40% acetonitrile in water with 0.1% FA) over 65 minutes using the Waters Nano UPLC system (room temperature, flow rate 500 nL/min), and effluent was electrosprayed into a LTQ mass spectrometer (Thermo Fisher Scientific). For the database search (Sequest), human Uniprot and NF1 protein sequences (variable modifications: phosphorylation [S, T, Y]) were used. Individual probability values for each putatively phosphorylated site were generated by Proteome Discoverer, version 1.3, equipped with the phosphoRS 2.0 node.

### TUBEs

Lysates from cells treated with si*FBXW11* or siControl were incubated with TUBEs-agarose (displays equivalent affinities for both K63 and K48 tetra-ubiquitin, TUBE 2) for 1 hour at room temperature (LifeSensors). TUBEs-agarose was washed after ubiquitin capture, and the bound material was eluted with Laemmi sample buffer. The relative levels of ubiquitylated neurofibromin were determined by immunoblotting with neurofibromin antibody (ab238142, Abcam) or ubiquitin antibody (no. 3933, Cell Signaling Technology).

### NanoBiT luciferase assays for GRDiso1/GRD1so2-FBXW11 interactions

GRD1/GRD2 and FBXW11 were amplified from cDNA of D3iso1/D3iso2 and then inserted into pFN33KLgBit-TK-neo Flexi, pFC34KLgBit-TK-neo Flexi, pFN35KSmBit-TK-neo Flexi, and pFC36KSmBit-TK-neo Flexi plasmids (Promega). HEK293T cells were transfected with the plasmids of NanoBiT fusion GRD1/GRD2 and FBXW11 constructs using FuGENE HD Transfection Reagent (Promega) according to the manufacturer’s protocols. Forty-eight hours after transfection in a 96-well plate, Nano-Glo Live Cell Substrate (Promega) was added to each well, incubated for 5 minutes, and an equal amount of reaction medium from each well was measured by Glo-MAX luminometer (Promega). Luciferase activities are shown as the mean value of triplicate wells ± SD.

### Ras activation assay

Brain tissues were homogenized in xTractor buffer (catalog 635671, TakaRa), and the protein lysates were collected after centrifugation at 13,000*g* for 10 minutes. Active Ras was pulled down with Raf-1 RBD agarose (catalog 14-278, EMDMillipore) overnight at 4°C with gentle agitation. After washing the beads with TBS, 3X NuPAGE LDS Sample buffer (catalog NP0007, Thermo Fisher Scientific) was added, and samples were boiled for 5 minutes. The supernatan was collected after brief centrifugation for immunoblotting to detect vinculin (catalog ab219649, Abcam) or Ras (catalog 05-516, EMDMillipore).

### Behavioral experiments

Once weaned on P28, all mice were group-housed (3–4/cage by litter containing mixed genotypes), given food and water ad libitum, and maintained on a 12-hour light/12-hour dark cycle (7 am/7 pm) at 72°F. Animals were single-housed either 1 week before the SP task or after surgery. For all behavioral tasks, the experimenters performing the behavioral task test and scoring the behavioral data were blinded to the genotype and treatment conditions.

### SP task

The 3-chamber SP task occurred over 2 days as described previously (15). After being single-housed for at least 1 week, testing began on day 1 when the mouse was placed in the center of the 3-chamber apparatus with 2 empty wire cups for 10 minutes of habituation (HAB). The mouse was blocked using clear Plexiglass in the center of the apparatus during the inter-trial interval of 5 minutes. The examiner placed a partner mouse under one of the wire cups before allowing the test mouse to investigate the partner mouse for 10 minutes of familiarization (FAM). During the inter-trial interval, the examiner placed another partner mouse under the second cup (novel 1 [NOV1]). For 10 minutes (NOV1), the test mouse was permitted to investigate the familiar mouse and NOV1 mouse. The examiner removed NOV1 during the inter-trial interval. To ensure familiarity, the test mouse then spent 45 minutes with the familiar mouse (FAM45) under the cup. Twenty-four hours later, the same familiar mouse was placed under the same wire cup, and a new novel mouse (NOV2) was placed under the other wire cup. The test mouse was introduced to the apparatus and given 10 minutes to investigate (NOV2). The time spent near each wire cup was recorded and scored with the automated AnyMaze software during the NOV1 and NOV2 time blocks.

### OF tests

Based on methods described previously, mice were placed in the dark in an open chamber (40 × 40 cm, 30 cm height) for 60 minutes while activity was recorded using AnyMaze software (14).

### DDT tests

Based on methods described previously, mice were trained to identify which side of a T maze (each arm measured 36 cm length, 8 cm width, and 12.5 cm height) had a large reward (4 Cocoa Krispies) compared with the small reward (1 Cocoa Krispie) (14). Training was completed daily with 6 trials per day with no delay until each mouse found the large reward correctly 10 out of 12 times over 2 consecutive days. After this, the delay was initiated. Testing consisted of 6 days with 6 trials per day, and teaching occurred on the first 2 days. The mouse was placed in the T maze with the small reward in place and no reward on the large reward side. The mouse would go to the large side as trained and would be blocked in this arm for teaching of the 10-second delay, and then the large reward would be given. After the first 2 days, which allowed for these teaching trials, the mice performed the task without being blocked in the large reward arm. Data were analyzed for the last 2 days for a total of 12 trials. For each testing trial, they could make 3 choices: directly go to small reward side, go to large reward side and then switch to the small reward side, or go to the large reward side and wait for the large reward (Figure 4A). A direct small choice or a switch choice would result in receiving a small reward (graphed in Figure 4C and Figure 5D). Mice were food-restricted during this time to increase food motivation, with daily measurement of weight and careful inspection of the health of each mouse.

### CAR tests

Based on previously described methods, a round, plastic platform (diameter, 20 cm; thickness, 2 cm) supported by a plastic rod (height, 50 cm) was used to assess the CAR (14). The platform was stabilized in a kiddie pool with a rubber bottom for cushion if the animal fell. Mice were placed in the center of the platform, and behavior was recorded for 60 minutes. If a mouse fell from the platform, it was immediately placed back on the platform, and the test continued up to 60 minutes. A mouse was considered to have an intact CAR if it did not fall off the platform. The CAR was calculated as a percentage of mice that demonstrated an intact CAR for each group: percentage of mice with an intact CAR = (the number of mice that did not fall from the platform/total number of mice) × 100. Head time in each zone was recorded and scored with the automated AnyMaze software. The edge zone was defined by outlining the outer 1 inch of the round platform with 1 inner circle and 1 outer circle in the AnyMaze software. The over-the-edge zone was defined by outlining an outer circle that was 2 inches from the edge of the platform in the AnyMaze software.

### Brain stereotaxic surgery

To excise *Fbxw11* in the tissue of interest, utilizing *Nf1^+/+^ Fbxw11^fl/fl^* and *Nf1^+/–^ Fbxw11^fl/fl^* genetically engineered mice, the experimenter performed stereotaxic surgery to inject either control (GFP; AAV5-CMV-GFP, UNC) or Cre-recombinase fused to GFP (CRE; AAV5-CMV-Cre-GFP, UNC) into brain tissue. Prior to all surgeries, mice were deeply anesthetized in an induction chamber using an isoflurane system (MGX Research Machine, Vetamic). Mice were then placed in a nose cone connected to the isoflurane system in the 900 series Ultraprecise Kopf Instruments stereotaxic apparatus for rodents. After ensuring flatness of the skull through measurement of lambda and bregma, bilateral viral transduction of the nucleus accumbens was performed (Figure 5A). Immediately following all stereotaxic surgeries, all mice received carprofen (5 mg/kg) and were observed over time, with carprofen (5 mg/kg) given at regular intervals until fully recovered. On the basis of data from previous studies (14, 15), we tested behavioral phenotypes before surgery and 8 weeks after surgery.

### Brain IHC

Mice were perfused transcardially with PBS, followed by 4% paraformaldehyde, and the brains were removed and coronally sectioned at 35 μm. After washes with PBS, H_2_O_2_, and PBS with Triton X-100 (PBST), the primary antibody diluted in PBST was applied to sections overnight. The next day, after washing 3 times with PBS, sections were placed with a secondary antibody in PBST for 90 minute and then amplified by avidin-biotin complex (ABC) (1:500, Vector Laboratories) in PBST for 90 minutes. After washing, Vector SG Substrate Kit (Vector Laboratories) was used to identify immunoreactivity. Sections were stored in phosphate buffer and mounted on slides with DAPI (Vectashield). Photomicrographs were taken (×200), and neurofibromin-immunoreactive (NF1-ir) cells and pERK-immunoreactive (pERK-ir) cells were counted by a blinded observer using ImageJ software (NIH). GFP-ir cells were used to quantify the injection targets.

#### Neurofibromin staining.

The primary antibody used was rabbit anti–neurofibromin polyclonal antibody (1:400, LSBio, LS-B8110), and the secondary antibody used was biotinylated anti-rabbit antibody (1:200, Vector Laboratories, BA-1000).

#### pERK staining.

The primary antibody used was rabbit anti-pERK polyclonal antibody (1:200, Cell Signaling Technology, no. 4695), and the secondary antibody used was biotinylated anti-rabbit antibody (1:200, Vector Laboratories, BA-1000).

#### GFP staining.

The primary antibody used was mouse anti-GFP polyclonal antibody (1:100, Life Sciences Technology, AB3080), and the secondary antibody used was anti–mouse Alexa Fluor 488 (1:200, Life Sciences Technology, A-11001).

### Statistics

All values are presented as the mean or percentage ± SEM unless otherwise indicated. All Western blot and in vitro assay experiments were performed at least in triplicate, and representative results are shown. Analysis was performed using GraphPad Prism version 5.0d (GraphPad Software). A *P* value of less than 0.05 was considered statistically significant unless stated otherwise. Baseline behavioral data for mice before surgical intervention was analyzed by 2-way ANOVA to ensure randomization. All behavioral tasks were analyzed in GraphPad Prism using 1- or 2-way ANOVA with genotype with or without treatment as the main factors, and the dependent variable being head time (s) near each partner mouse cup (3-chamber SP task), total distance traveled (cm, OF), zone time (s) or entries (CAR), and the number of small impulsive choices made (DDT). For brain IHC, neuron counts were analyzed by 2-tailed *t* test or 1-way ANOVA with viral genotype with or without treatment as the main factors. If a factor or interaction was significant, we ran a Tukey’s or Dunnett’s multiple-comparison post hoc test as indicated in the figure legends.

### Study approval

Animal care procedures were conducted in accordance with the NIH *Guidelines for the Care and Use of Laboratory Animals* (NIH Publication No. 80-23; National Academies Press, 2011) revised 1996, with procedures approved by the Indiana University School of Medicine IACUC (protocol no. 19045, 21009).

### Data availability

No analytic code was generated for this study. Supporting data values are available in the Supporting Data Values file as a single Excel file with multiple tabs. All other data associated with this study can be provided by the corresponding author upon request.

## Author contributions

SJP, JLL, KKC, and DWC designed research studies. SJP, JLL, KKC, HPD, CBB, SQ, MMS, CGG, NC, SA, MAC, JZ, JHW, AS, and LJ conducted experiments. SJP, JLL, KC, HPD, and SPA analyzed data. SJP, JLL, KC, SPA, and DWC wrote the manuscript. SJP, JLL, KKC, DKM, SDR, SPA, and DWC edited the manuscript. JLL, SPA, and DWC acquired funding. JLL, SPA, and DWC supervised the study. SJP, JLL, and KKC are designated as co–first authors due to their unique but equal contributions to this work and the listed order was chosen on the basis of the timing of their involvement. SJP initiated the genetic screening, biochemical, and in vitro assays; JLL supervised the neurodevelopmental murine studies; and KKC performed biochemical assays and murine tissue analyses.

## Supplementary Material

Supplemental data

Unedited blot and gel images

Supporting data values

## Version 1 07/01/2025

Electronic publication
