# Supplementary material for: A haploinsufficiency restoration strategy corrects neurobehavioral deficits in Nf1+/– mice
Source: J Clin Invest. 2025 Jul 1;135(13):e188932. doi: 10.1172/JCI188932 (PMC12208548; doi:10.1172/JCI188932)
Supplement: Supplemental data [file jci-135-188932-s043.pdf]

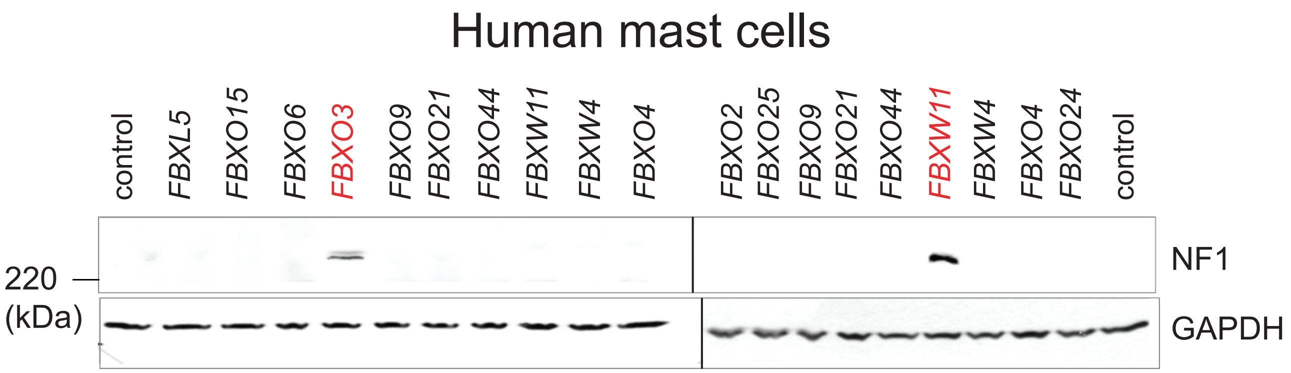

Supplemental Fig. 1. From the F-box library, each F-box specific siRNA was transfected into the human mast cell line LUVA. Seventy-two hours post-transfection, cells were harvested and lysates were used for detection of neurofibromin by immunoblotting.

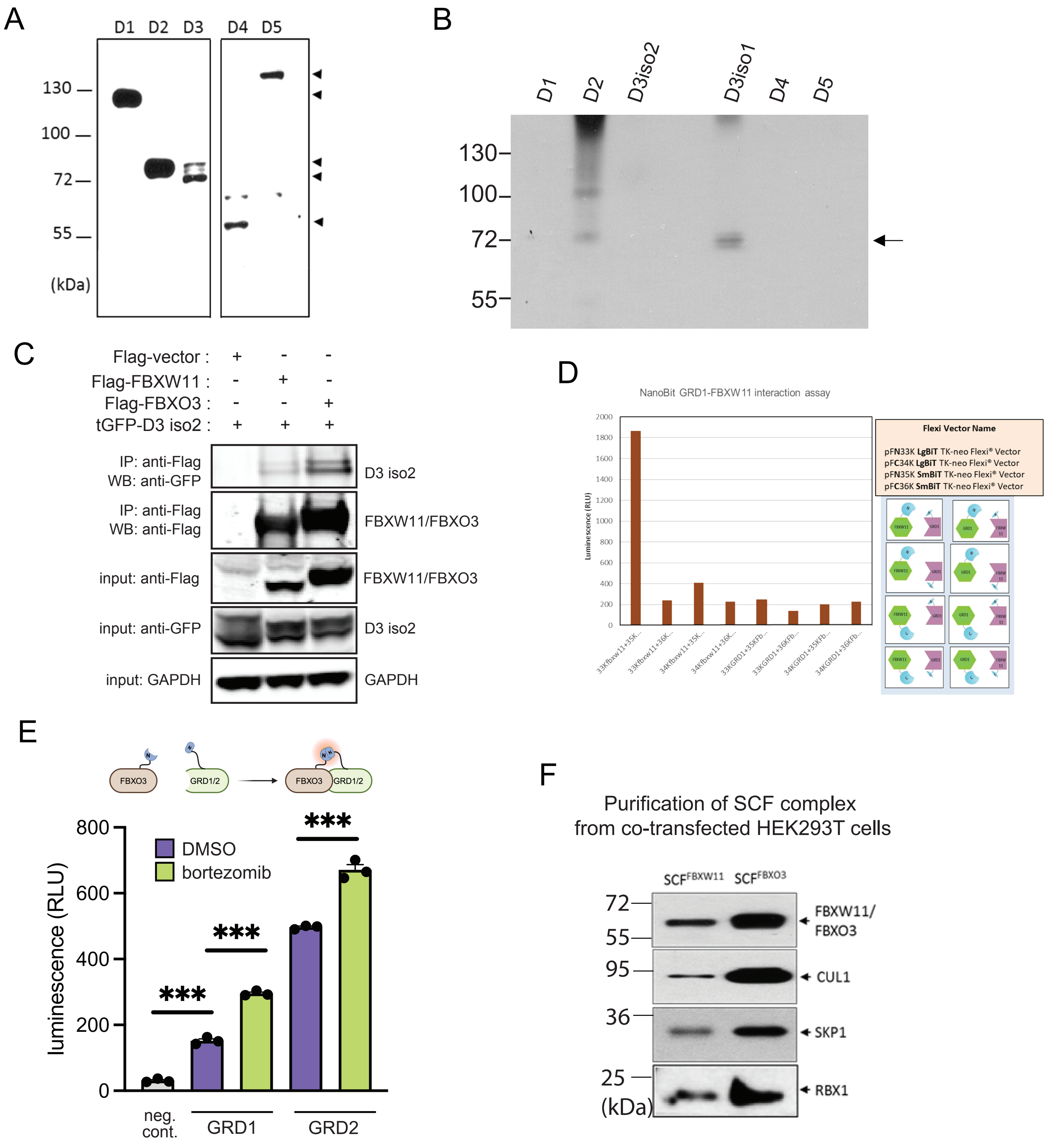

Supplemental Fig. 2. (**A**) Western blotting of the fragments with anti-GST to detect neurofibromin constructs. (**B**) HEK293T cells were harvested and subjected to co-immunoprecipitation using anti-Flag monoclonal antibody to bind Flag-tagged FBXW11 and the precipitated proteins were analyzed by western blotting using antibodies against tGFP. (**C**) HEK293T cells transfected with GFP-tagged domain 3 of neurofibromin isoform 2 (GRD2) and either empty Flag, Flag-FBXO3, or Flag-FBXW11 were treated with 15 μM MG-132 six hours prior to harvest, then input or Flag immunoprecipitation was performed prior to immunoblotting. Normalization of each D3 iso2 pulldown to the corresponding FBX IP (GFP/Flag ratio) showed that FBXO3-iso2 binding was 2.7 fold higher than FBXW11-iso2. (**D**) Orientation screen using eight different plasmid combinations compared by NanoBit signal to detect and optimize GRD1 and FBXW11 signal. (**E**) Nano-BiT complementation assay in HEK293T cells using Lg-BiT-FBXO3 and SmBiT-GRD1 or GRD2 with vehicle or 1 μM bortezomib. (**F**) Western blot analysis of SCF-FBXW11/FBXO3 complex with co-IP using anti-Flag antibody (Flag-tagged FBXW11 or FBXO3). In lieu of stripping and reprobing, the same samples were run on a separate gel to detect RBX1 in this figure. Image created in BioRender. Angus, S. (2025) https://BioRender.com/x35a350.
