# Supplementary material for: A haploinsufficiency restoration strategy corrects neurobehavioral deficits in Nf1+/– mice
Source: J Clin Invest. 2025 Jul 1;135(13):e188932. doi: 10.1172/JCI188932 (PMC12208548; doi:10.1172/JCI188932)
Supplement: Unedited blot and gel images [file jci-135-188932-s044.pdf]

Full unedited blot/gel
for Fig. 1A - NF1

1 2
3 4 layout
5 6
7 8

1

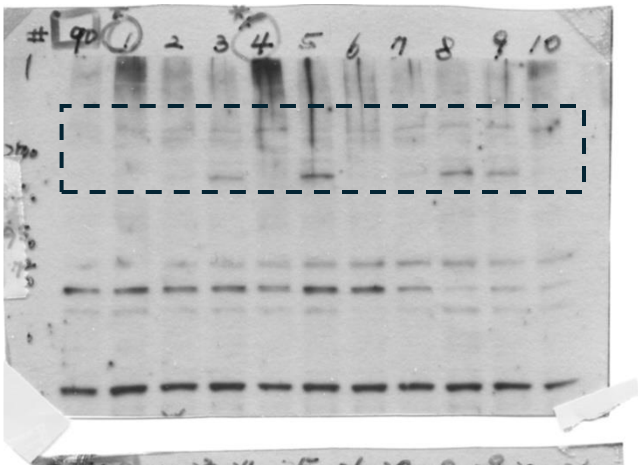

2

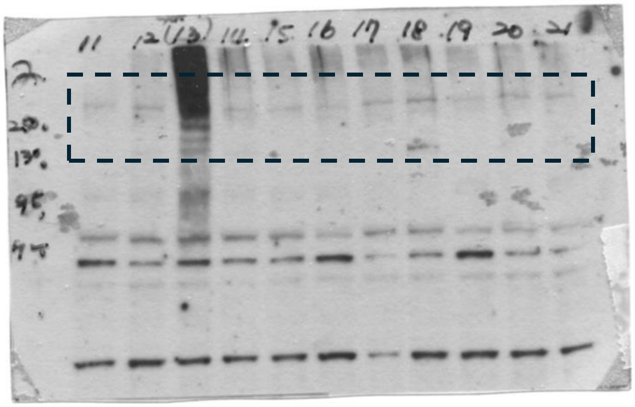

3

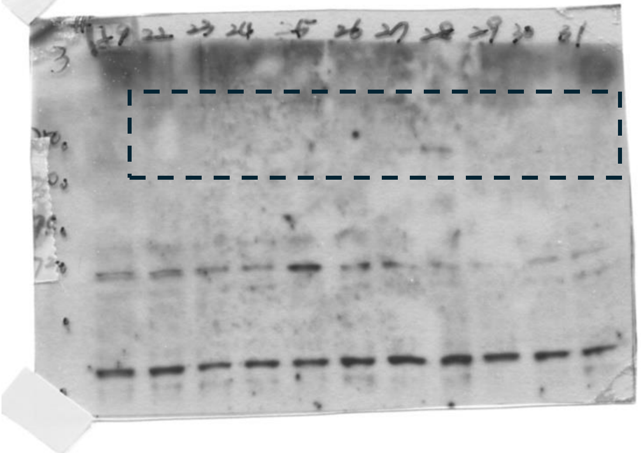

4

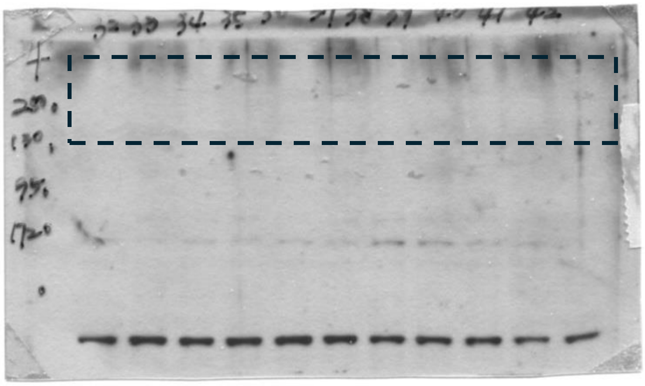

5

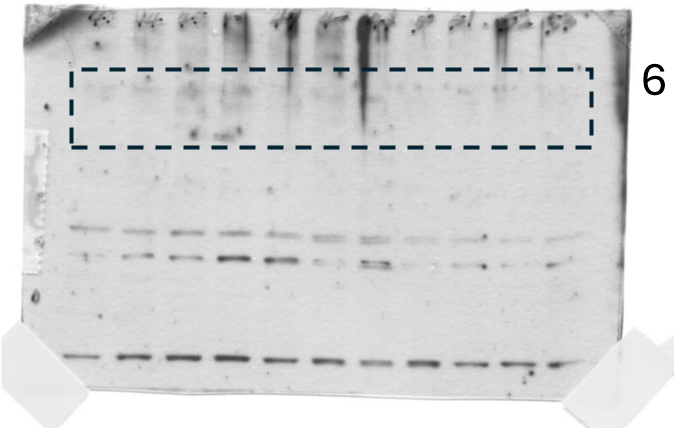

6

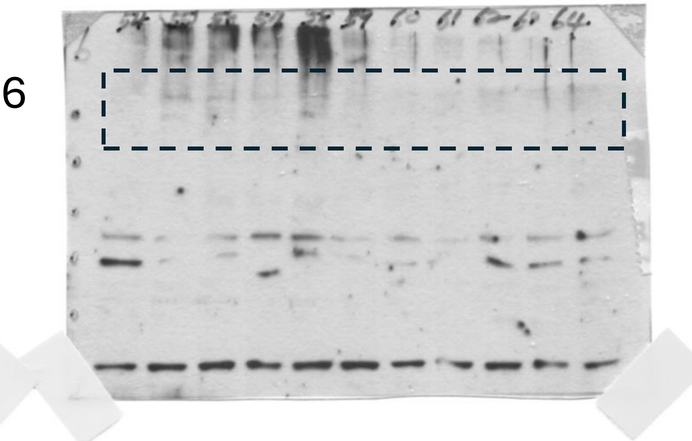

7

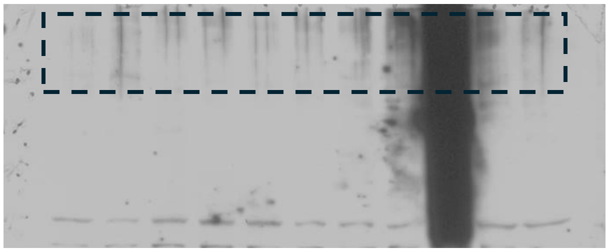

8

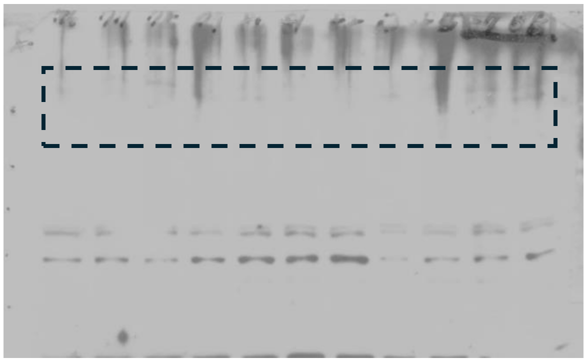

Full unedited blot/gel
for Fig. 1B - pERK1/2

1 2
3 4
5 6
7 8

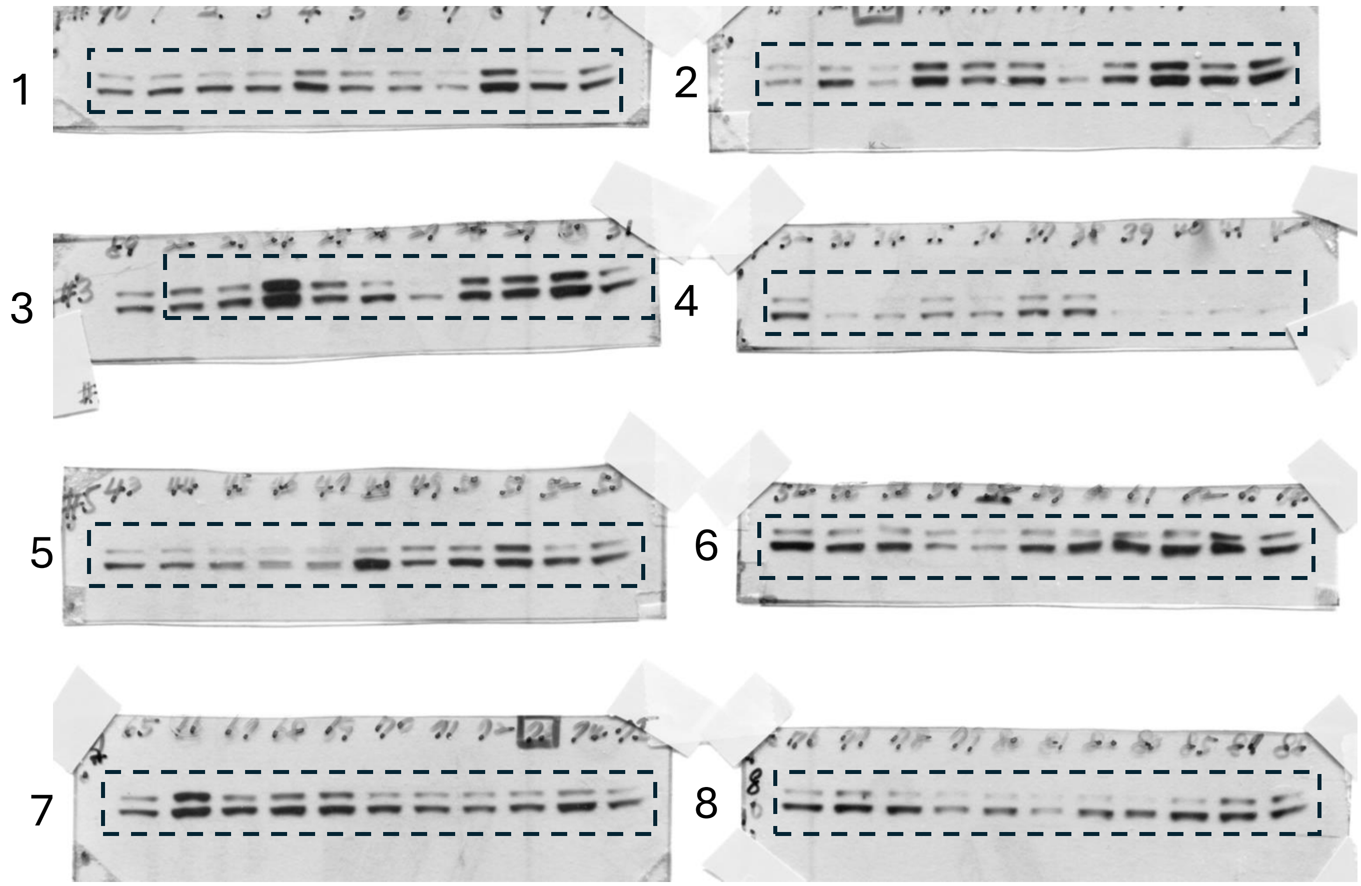

Full unedited blot/gel
for Fig. 1B

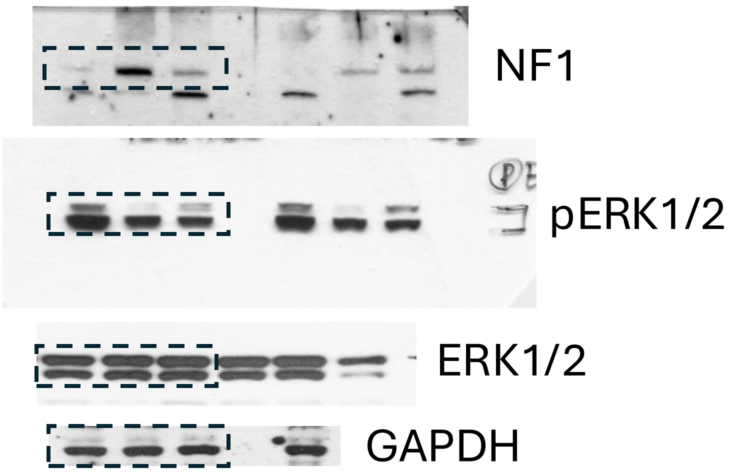

Full unedited blot/gel
for Fig. 1C

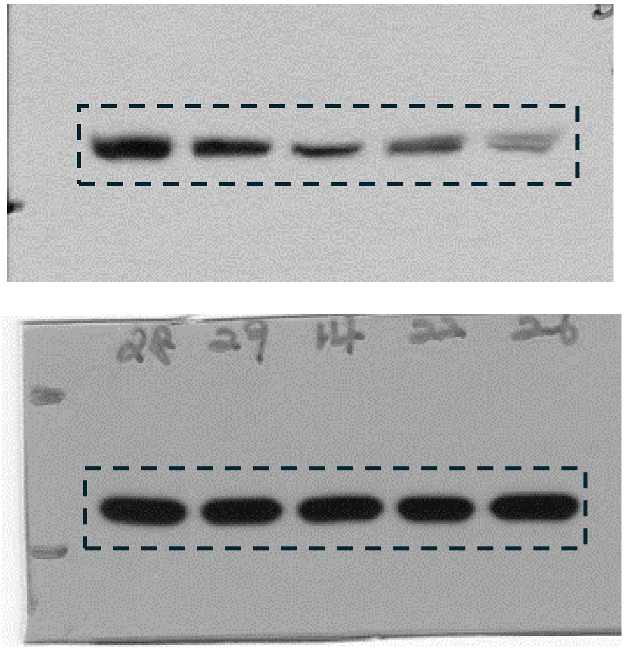

NF1

GAPDH

Full unedited blot/gel
for Fig. 1D

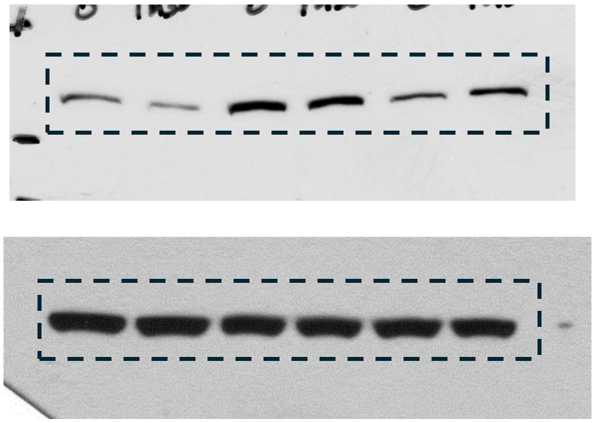

NF1

GAPDH

Full unedited blot/gel
for Fig. 2A

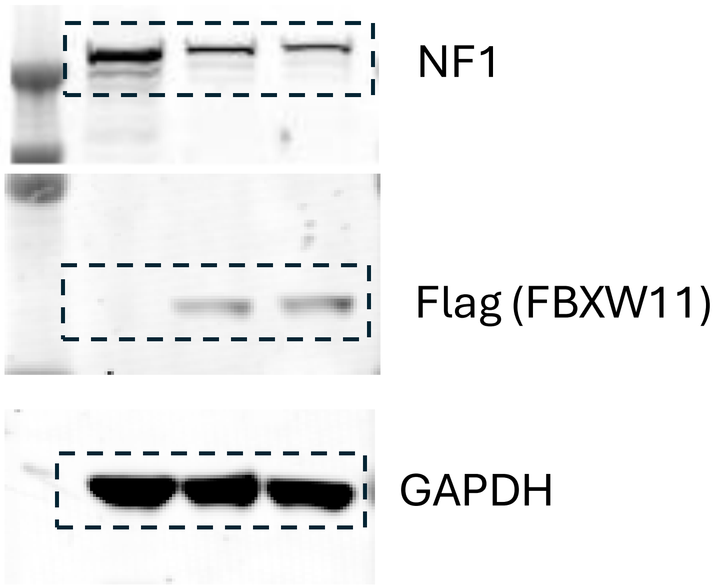

Full unedited blot/gel
for Fig. 2B

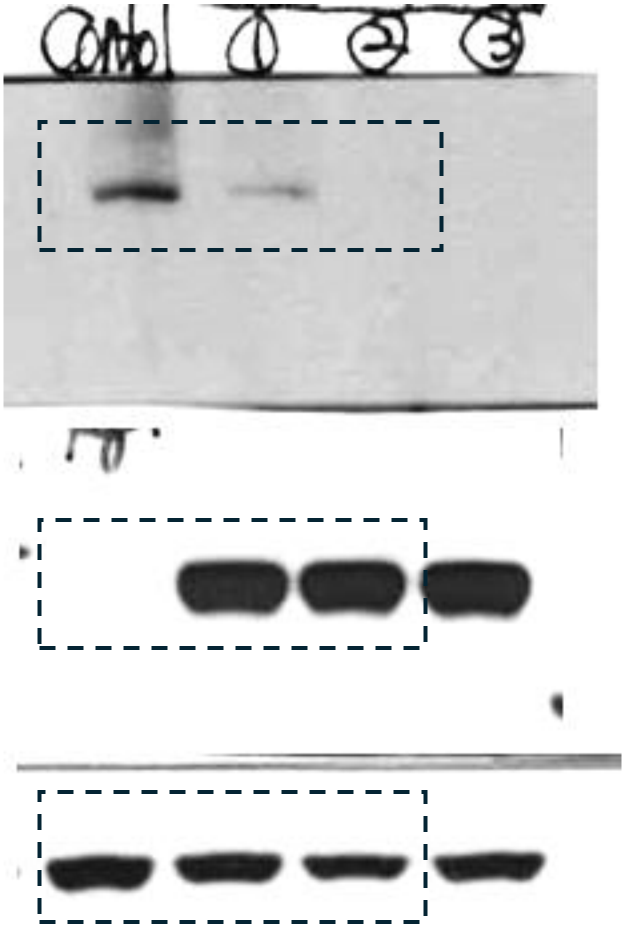

NF1

Flag (FBXO3)

GAPDH

Full unedited blot/gel for Fig. 2C

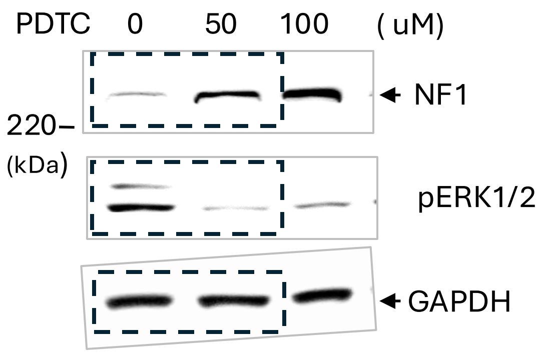

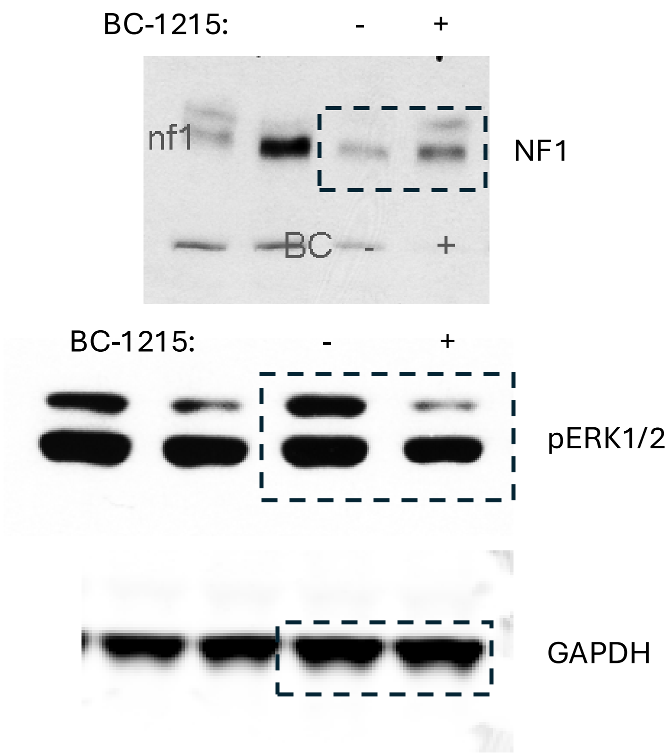

Full unedited blot/gel
For Fig. 3B

IP: anti-Flag
WB: GFP (D3 iso1)

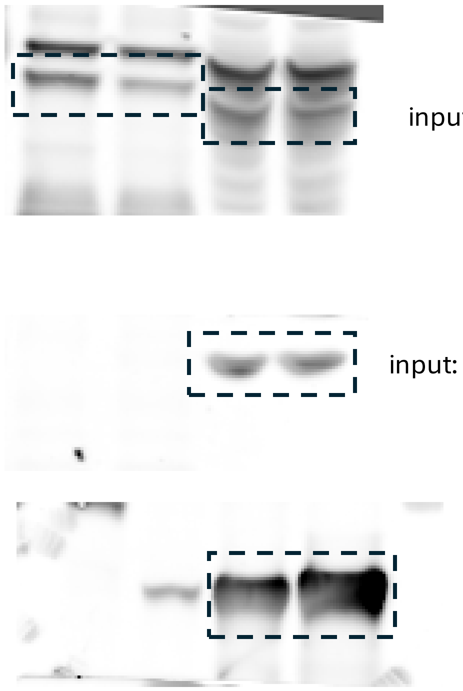

input: D3 iso1 (GFP)

input: GAPDH

IP: anti-Flag
WB: FBXW11/FBXO3 (Flag)

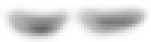

Full unedited blot/gel
For Fig. 3D

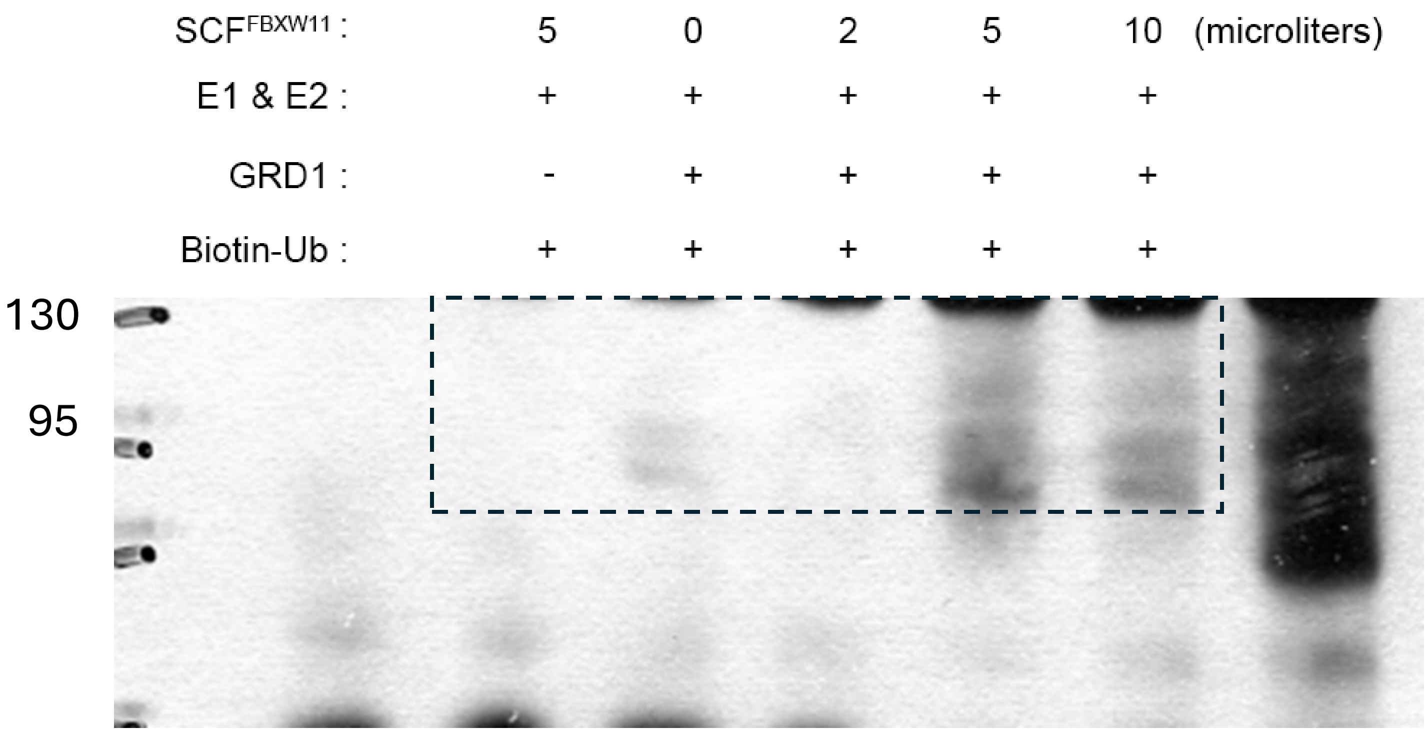

Full unedited blot/gel
For Fig. 3E

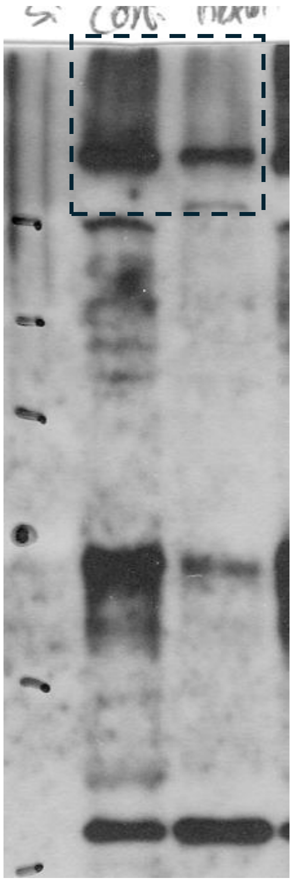

Poly-ub NF1

Cont siFbxw11

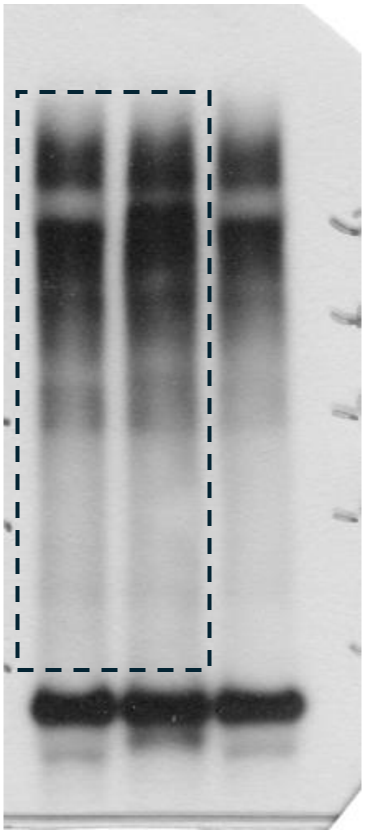

Poly-ub
Total protein

Full unedited blot/gel
For Fig. 4C

IP: anti-GFP
WB: anti-Flag

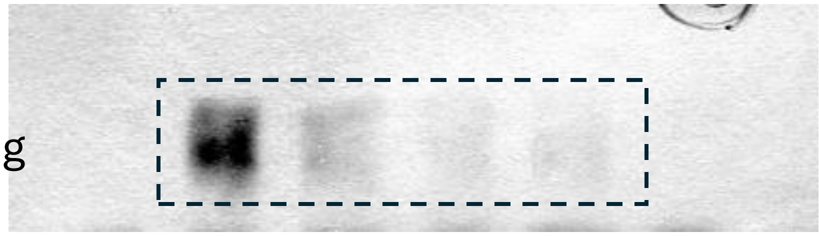

FBXW11

input

FBXW11

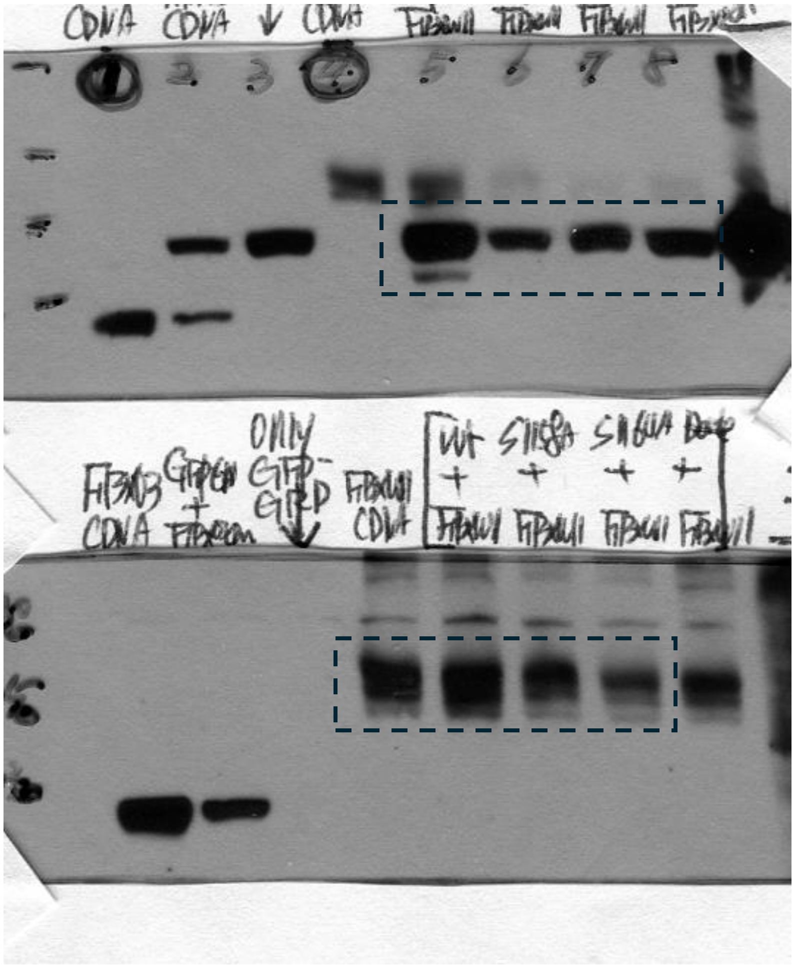

input

GRD1

Full unedited blot/gel
For Fig.8C

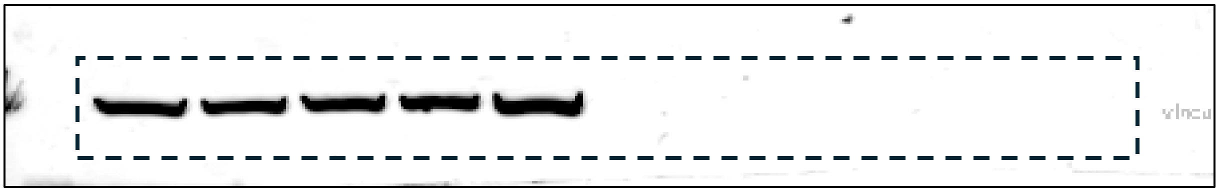

Vinculin

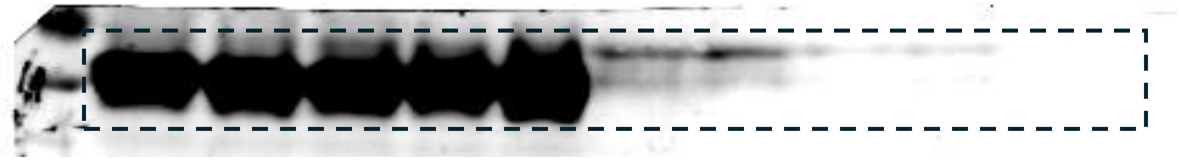

Ras

Full unedited blot/gel

For Supp. Fig.1

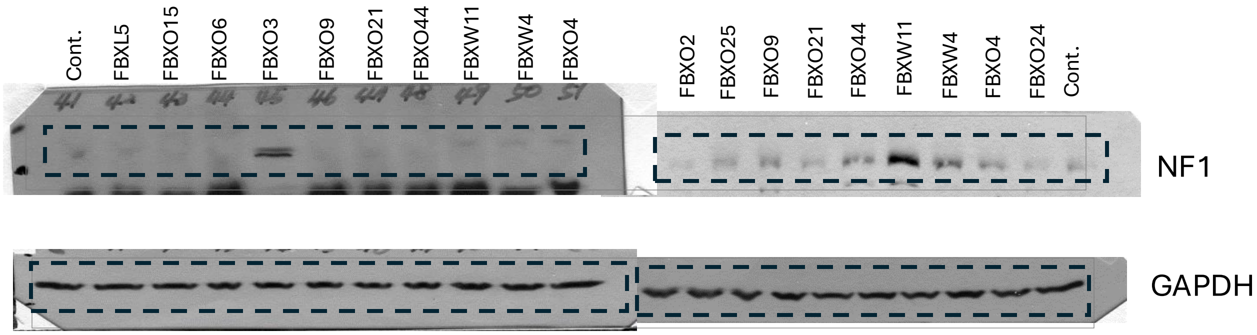

Full unedited blot/gel
For Supp. Fig.2A

D1 D2 D3

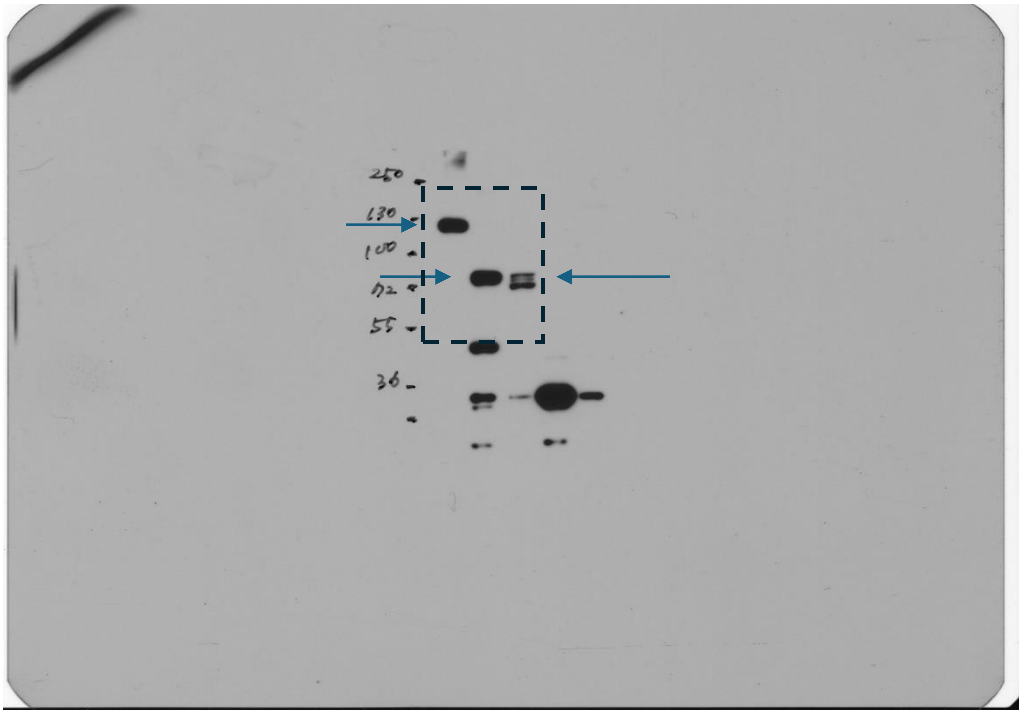

D4 D5

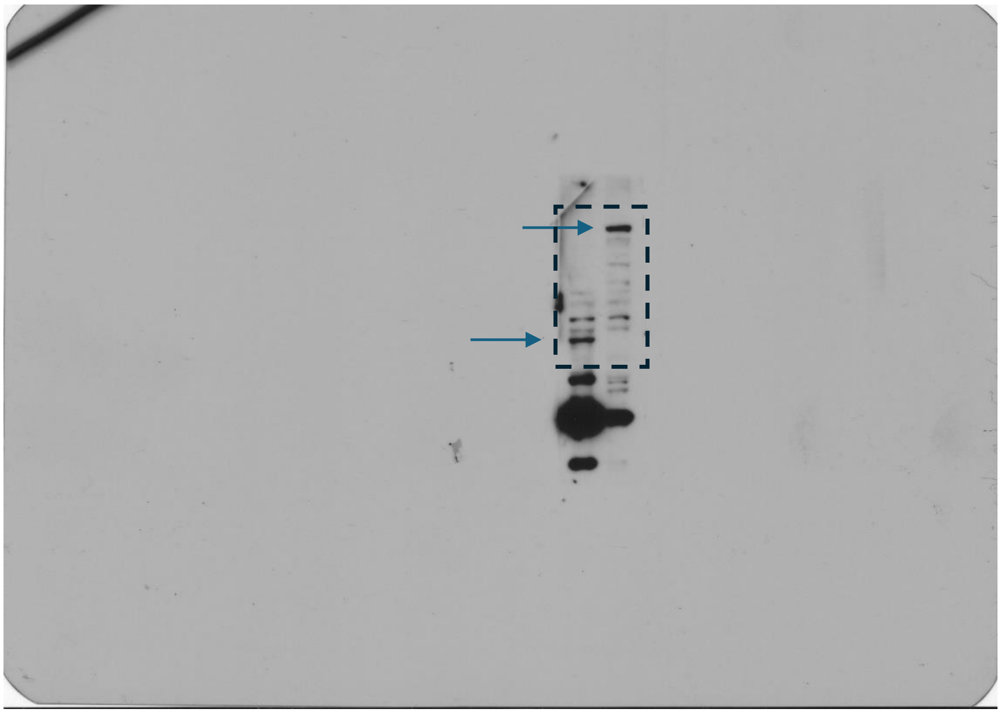

Full unedited blot/gel
For Supp. Fig.2B.

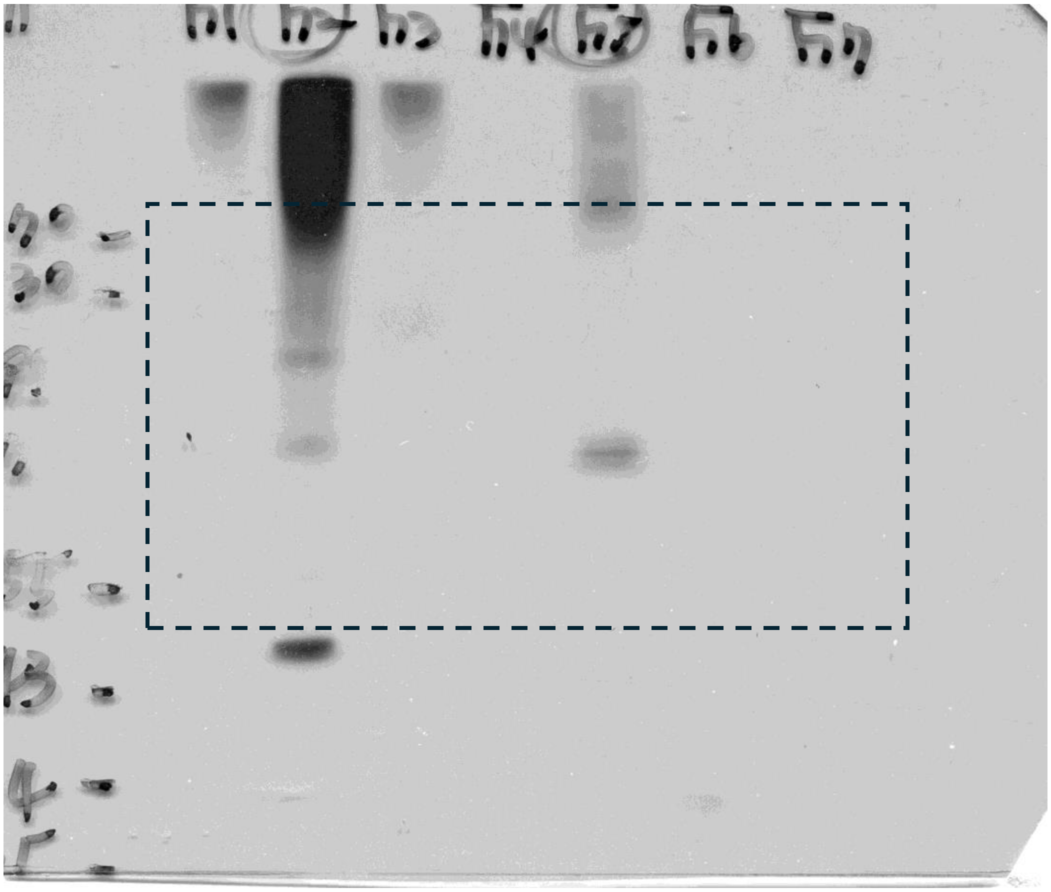

Full unedited blot/gel
For Supp. Fig.2C

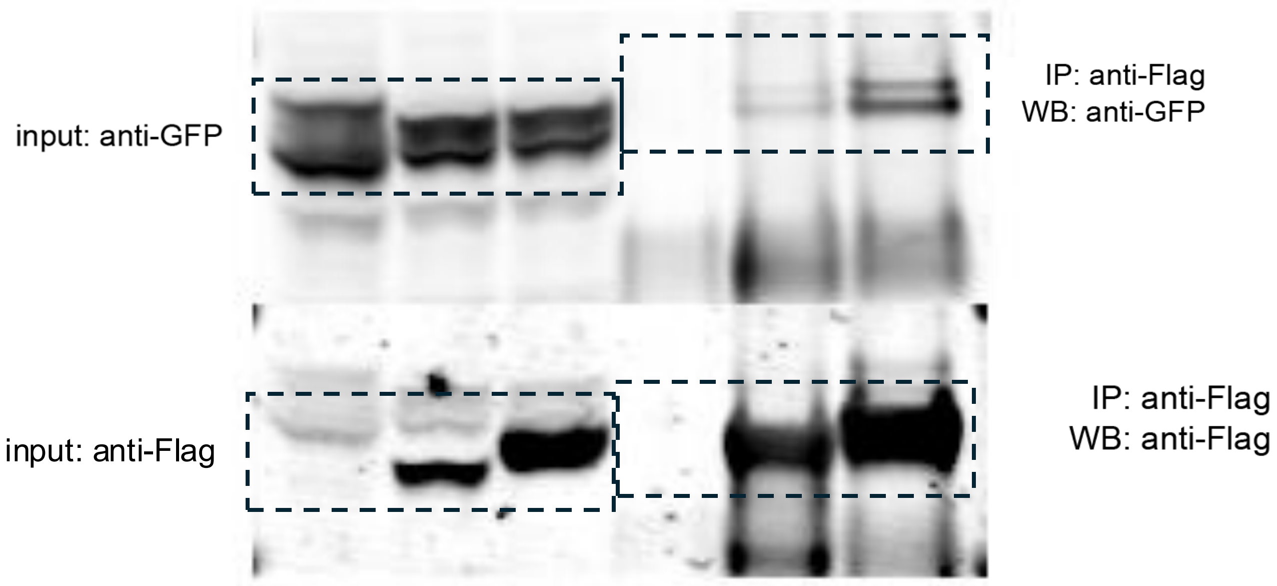

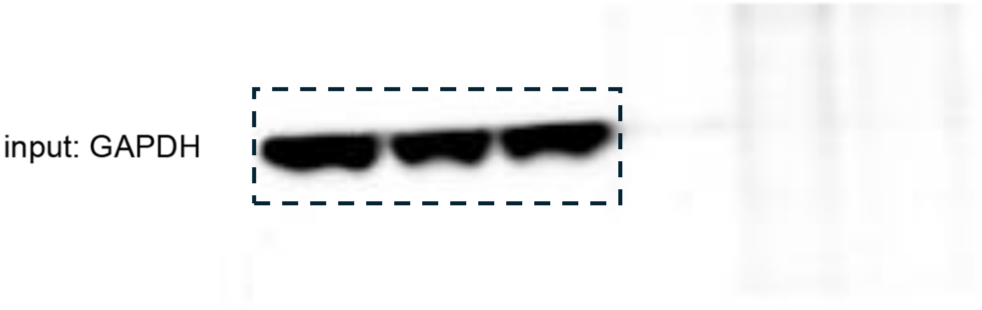

Full unedited blot/gel
For Supp. Fig.2F

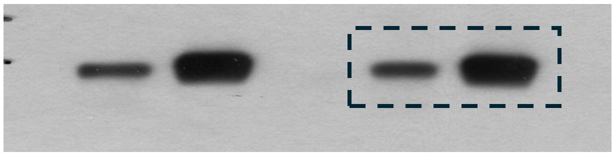

FBXW11, FBXO3

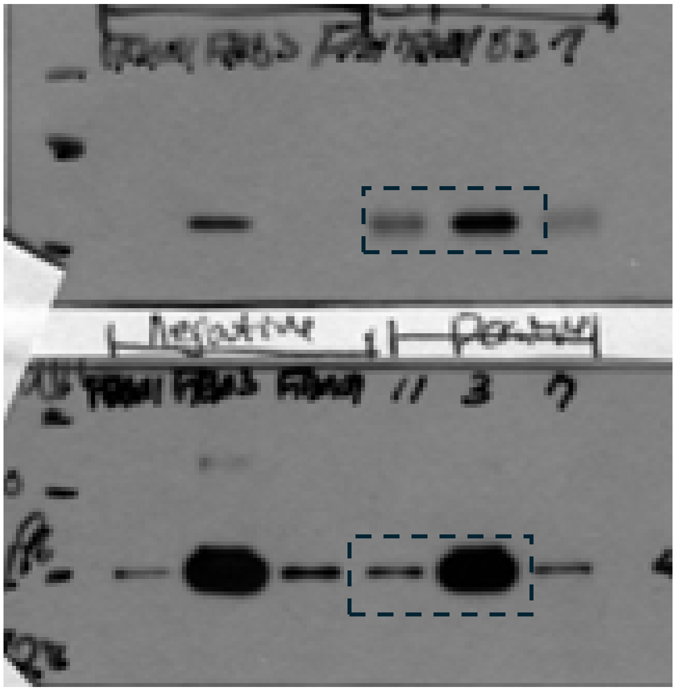

SKP1

CUL1

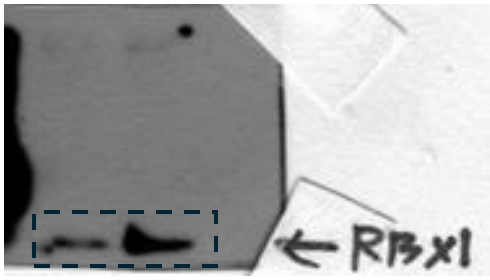

RBX1
